# TMEM92 shields DDX3X from TTC3‐mediated degradation to confer chemoresistance in triple‐negative breast cancer

**DOI:** 10.1002/ctm2.70681

**Published:** 2026-05-15

**Authors:** Hao Shen, Xiaochao Jia, Xu Li, Zhi Li, Zhihua Zhang, Yang Zhao, Lei Shen, Xiaoqiu Bu, Qiang Ma, Chunli Liang, Xiaoti Lin, Lin‐Xiaoxi Ma, Chuan Qin

**Affiliations:** ^1^ Department of Thyroid Breast Surgery Shanghai East Hospital, School of Medicine, Tongji University Shanghai China; ^2^ Department of Medical Ultrasound Jinshan Hospital of Fudan University Shanghai China; ^3^ Center of Hepatobiliary Pancreatic Disease Xuzhou Central Hospital Xuzhou Jiangsu China; ^4^ Department of Breast and Thyroid Surgery The Affiliated Huaian No. 1 People's Hospital of Nanjing Medical University Huai'an Jiangsu China; ^5^ Department of Breast Surgery Fudan University Shanghai Cancer Center, Key Laboratory of Breast Cancer in Shanghai Shanghai China; ^6^ Department of Oncology Shanghai Medical College Fudan University Shanghai China

**Keywords:** cisplatin resistance, DDX3X, TMEM92, triple‐negative breast cancer, TTC3, ubiquitination

## Abstract

**Background:**

Triple‐negative breast cancer (TNBC) remains a major clinical challenge because of its aggressive characteristics, limited targeted treatment options, and frequent chemoresistance. However, the molecular mechanisms governing protein stability that drive TNBC progression and therapeutic resistance remain incompletely understood.

**Methods:**

TMEM92 expression and clinical relevance were evaluated using public datasets, patient specimens, and TNBC cell models. Loss‐of‐function, rescue, xenograft, protein interaction, and ubiquitination assays were performed to determine the biological function and molecular mechanism of TMEM92 in TNBC progression and cisplatin response.

**Results:**

TMEM92 was prominently expressed in TNBC and correlated with poor prognosis. Functionally, depletion of TMEM92 suppressed TNBC cell proliferation, migration, invasion, and survival while promoting apoptosis in vitro and in vivo. Mechanistically, TMEM92 directly associated with DEAD‐box helicase 3 X‐linked (DDX3X) and protected it from degradation by the E3 ubiquitin ligase tetratricopeptide repeat domain 3 (TTC3). TMEM92 competitively prevented TTC3 binding to DDX3X, thereby inhibiting TTC3‐mediated K48‐linked ubiquitination and subsequent proteasomal degradation of DDX3X. Re‐expression of DDX3X rescued the anti‐tumor effects induced by TMEM92 knockdown. Therapeutically, TMEM92 targeting sensitized TNBC cells and xenograft tumors to cisplatin. TMEM92 knockout reduced the cisplatin IC_50_ by 44.0% in MDA‐MB‐231 cells and 42.9% in BT‐549 cells, and TMEM92 depletion enhanced cisplatin‐induced tumor growth inhibition by approximately 70.6% compared with cisplatin alone.

**Conclusions:**

This study identifies a novel TMEM92DDX3XTTC3 axis that regulates DDX3X protein stability and drives TNBC progression and chemoresistance, revealing a potential prognostic and therapeutic vulnerability in TNBC.

## Key points

- TMEM92 functions as an oncogenic driver and prognostic marker in triple‐negative breast cancer.
- TMEM92 shields DDX3X from TTC3‐dependent K48‐linked ubiquitination to sustain tumor cell survival.
- Disruption of the TMEM92DDX3X axis sensitizes TNBC cells to cisplatin treatment.

## Graphical abstract

TMEM92 protects DDX3X from degradation by the E3 ligase TTC3, promoting TNBC cell proliferation and tumour growth. Depleting TMEM92 not only suppresses tumour growth but also sensitises cells to cisplatin, suggesting that targeting this axis is a potential therapeutic strategy.

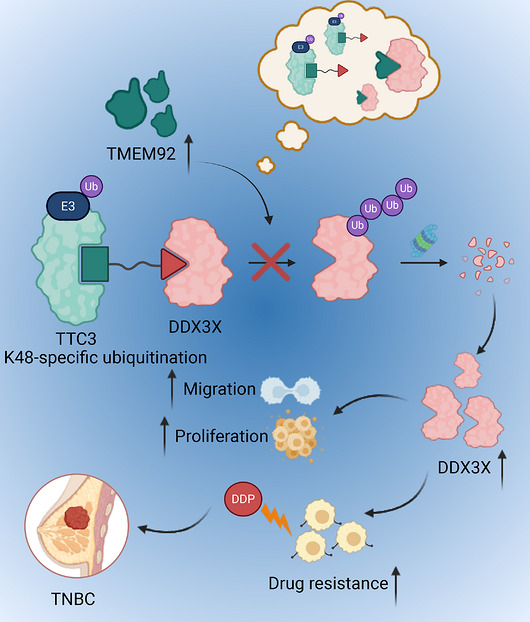

## 1 INTRODUCTION

Triple‐negative breast cancer (TNBC) stands out as one of the most arduous subtypes of breast cancer in a clinical context. It is distinguished by its aggressive nature, a substantial likelihood of spreading to other parts of the body (metastasis) and an unusually unfavourable prognosis.^1^, ^2^, ^3^ Epidemiologically, TNBC accounts for 10–15% of incident breast cancers and carries the worst prognosis.^4^ TNBC is characterised by the absence of oestrogen receptor (ER), progesterone receptor (PR) and human epidermal growth factor receptor 2 (HER2) expression. As a result, TNBC is inherently insensitive to endocrine therapy and HER2‐directed treatments that are effective in other breast cancer subtypes.^5^, ^6^, ^7^ Consequently, the therapeutic armamentarium for TNBC primarily relies on cytotoxic chemotherapy, with platinum‐based agents like cisplatin (DDP) being a cornerstone of treatment, particularly for patients with germline *BRCA* mutations.^8^, ^9^ Mechanistically, DDP exerts its anti‐tumour effects mainly by forming DNA intra‐ and inter‐strand crosslinks, thereby inducing replication stress, DNA damage and apoptosis in rapidly proliferating cancer cells. However, due to its lack of tumour specificity, cisplatin can also damage normal proliferating tissues, leading to dose‐limiting toxicities such as nephrotoxicity, neurotoxicity and myelosuppression.^10^ However, the clinical utility of these agents is severely hampered by the frequent emergence of intrinsic and acquired chemoresistance, leading to high rates of treatment failure and disease recurrence.^11^, ^12^, ^13^ This stark clinical reality underscores a pressing need to identify novel molecular drivers of TNBC pathogenesis that can be exploited as new therapeutic targets.

Transmembrane (TMEM) proteins constitute a large and diverse superfamily, many of which act as receptors, channels or signalling scaffolds that are frequently dysregulated in cancer.^14^, ^15^, ^16^ For instance, TMEM16A (ANO1) has been reported to promote proliferation and invasion in TNBC through calcium‐activated chloride channel activity and downstream EGFR signalling,^17^ while TMEM45A has been linked to hypoxia adaptation and chemoresistance in basal‐like breast cancer.^18^ In contrast, TMEM92 remains largely understudied in breast cancer. To date, there are no systematic mechanistic studies elucidating its role in TNBC progression or chemotherapy response.^19^ Given that TMEM92 encodes a multi‐pass TMEM protein with potential roles in intracellular signalling and protein–protein interactions, we hypothesised that TMEM92 may represent a previously unrecognised regulator of chemoresistance in TNBC. Despite this, the biological functions of many TMEM family members remain unknown.^20^, ^21^ TMEM92 is one such protein, with only sparse reports linking it to cellular processes and no established role in oncology.^22^, ^23^ Our preliminary bioinformatics analysis of publicly available databases indicated that TMEM92 expression is significantly elevated in TNBC and correlates with unfavourable patient outcomes, suggesting it may be an unappreciated player in this disease.

The X‐linked DEAD‐box helicase 3 (DDX3X) belongs to the family of DEAD‐box RNA helicases and plays fundamental roles in RNA metabolism under physiological conditions. It is involved in multiple cellular processes, including mRNA transcription, splicing, nuclear export, translation initiation and stress granule assembly, thereby contributing to the maintenance of RNA homeostasis and cellular stress responses. In addition to these fundamental functions, accumulating evidence has linked DDX3X to therapy response in cancer. Prior studies have suggested that altered DDX3X stability or activity can influence chemosensitivity, including in TNBC, and that pharmacological inhibition of DDX3X may enhance the response to platinum‐based treatment, supporting a context‐dependent role for DDX3X in treatment resistance and stress adaptation.^24^, ^25^ In this study, we reveal that TMEM92 is a potent oncogenic driver that promotes TNBC progression by orchestrating a novel protein stabilisation mechanism. We demonstrate for the first time that TMEM92 directly binds to DDX3X and competitively inhibits its ubiquitination and degradation by the E3 ligase tetratricopeptide repeat domain 3 (TTC3). This stabilisation of the pro‐tumourigenic DDX3X protein is critical for TNBC cell proliferation, survival and migration. Crucially, we show that targeting the TMEM92–DDX3X axis profoundly sensitises TNBC cells to DDP. Consequently, our discoveries reveal a regulatory axis that was previously undetected that contributes to TNBC pathogenesis and represents a promising therapeutic vulnerability.

## 2 MATERIALS AND METHODS

### 2.1 Cell culture, antibodies and reagents

TNBC cell lines MDA‐MB‐231 and BT‐549, and the non‐tumorigenic breast epithelial cell line MCF‐10A, were obtained from ATCC (Manassas, VA, USA). MDA‐MB‐231 and BT‐549 cells were cultured in DMEM (Gibco, #C11995500BT) supplemented with 10% fetal calf serum (Gibco, #10099141C) and 1% penicillin‐streptomycin solution (Gibco, #15140122). MCF‐10A cells were cultured in DMEM/F‐12 medium (Gibco, #C11330500BT) supplemented with 5% equine serum (Gibco, #16050122), 20 ng/mL epidermal growth factor (PeproTech, #AF‐100‐15), 0.5 µg/mL hydrocortisone (Sigma‐Aldrich, #H0888), 100 ng/mL cholera toxin (Sigma‐Aldrich, #C8052), and 10 µg/mL insulin (Sigma‐Aldrich, #I3536). All cells were maintained at 37°C in a humidified incubator with 5% CO_2_.

TMEM92 (Abcam; #ab185545, 1:1000), DDX3X (Cell Signaling Technology (CST); #2635, 1:1500), TTC3 (Proteintech; #10915‐1‐AP, 1:800), HER2 (CST; #2165, 1:1000), ERα (CST; #8644, 1:1000), PR (CST; #8757, 1:1000), Ki67 (CST; #9449, 1:2000), Glyceraldehyde‐3‐phosphate dehydrogenase (GAPDH) (CST; #5174, 1:5000), Hemagglutinin (HA)‐tag  (CST; #3724, 1:1000), Flag‐tag (Sigma–Aldrich; #F1804, 1:2000), His‐tag (CST; #12698, 1:1000) and Myc‐tag (CST; #2276, 1:1000). Secondary antibodies coupled with Horseradish peroxidase (HRP) were sourced from CST and employed as per the instructions supplied by the manufacturer. DDP, MG132, chloroquine (CQ) and cycloheximide (CHX; 10 µg/mL) were all from MedChemExpress (MCE): #HY‐17394, #HY‐13259, #HY‐17589 and #HY‐12320.

### 2.2 Plasmid construction, shRNAs and lentivirus transfection

Full‐length human TMEM92, DDX3X and TTC3 complementary DNA (cDNAs) were cloned into pcDNA3.1 expression vectors carrying Flag, HA or Myc epitope tags. TMEM92 deletion mutants and His‐Ubiquitin plasmids were generated by mutagenesis. For lentivirus production, the following shRNA targeting TMEM92: shTMEM92‐1 and shTMEM92‐2 and TTC3: shTTC3‐1 and shTTC3‐2 and a non‐targeting control shNC were inserted into the pLKO.1 vector. The shRNA sequences can be found in Table S1. Lentivirus particles were produced in HEK‐293T cells, and then lentivirus particles were used to infect TNBC cells. After that, the infected cells were selected with puromycin (2 µg/mL) to establish stable cell lines. Generate TMEM92 knockout (KO) cell lines using CRISPR/Cas9 genome editing techniques. Cloned single‐guide RNAs targeting TMEM92 into a Cas9 expressing vector, then after the antibiotic selection and single cell cloning. TMEM92 KO was confirmed with Western blot. Two different KO clones were used in the following experiments. A non‐targeting single‐guide RNA was used as a negative control for CRISPR/Cas9‐mediated genome editing. All constructs were validated by knockdown and overexpression efficiencies with quantitative real‐time PCR (qRT‐PCR) and Western blot (Figure S1).

### 2.3 Quantitative real‐time PCR

Total RNA was isolated with TRIzol reagent (Invitrogen; 15596026) following the standard protocol.^26^ cDNA was synthesised using the PrimeScript RT Reagent Kit (Takara; #RR037A). RT‐PCR was performed on a QuantStudio 5 RT‐PCR System using SYBR Green Master Mix (Applied Biosystems; #A25742). Relative gene expression levels calculate using the 2^−ΔΔCt^ method, with GAPDH serving as the internal reference gene.^27^

### 2.4 Cell proliferation assay

Cell proliferation was evaluated using the Cell Counting Kit‐8 (CCK‐8; Dojindo; #CK04) following manufacturer's protocol. Briefly, 2000 cells were seeded per well in 96‐well plates. CCK‐8 reagent was added to each well, and the plates were incubated at 37°C for 2 h. After waiting for the incubation time, read the amount of light at 450 nm using a MicroPlate Reader.

### 2.5 Colony formation assay

Cells were seeded at 800 cells/well on six‐well plate and incubated for 10–14 days. Culturing medium was changed every day for 3 days. When the incubation period was over, the formed colonies were washed with PBS. Then, we used 4% paraformaldehyde for 15 min to fix the colonies, and then stained with a .1% crystal violet solution for 20 min. We did another wash and the plates were air dried. At last, we took some photos of those colonies which were visible and then counted the colonies.

### 2.6 Apoptosis assay

Cellular apoptosis levels were determined via flow cytometric analysis of double‐stained samples using reagents from BD Biosciences (#556547). Briefly, all treated cells – including both unattached and substrate‐bound populations – were collected, pelleted and reconstituted in a specific binding matrix. The cell suspensions were subjected to a 15‐min ambient incubation with the aforementioned fluorescent dyes under light‐deprived conditions. The percentages of cells undergoing early and late apoptosis were immediately recorded using a Beckman Coulter CytoFLEX instrument.

### 2.7 Wound healing assay

The directional migration potential of the cells was determined by culturing them to a densely packed state within six‐well plates, followed by mechanical disruption of the cell layer using a plastic 200‐µL tip. To prevent cell division from confounding the migration results, the wounded layers were washed with PBS to extract floating fragments and then incubated in a serum‐deprived culture medium. The scratch regions were photographed using an inverted microscope at exactly the same coordinates at 0 and 24 h. The cell‐free areas were digitally analysed with ImageJ software. Migration ability was ultimately evaluated by calculating the percentage of the initial scratched zone that became occupied by migrating cells.

### 2.8 Transwell migration and invasion assays

Cell migratory and invasive capacities were assessed using 24‐well Transwell inserts equipped with 8.0‐µm polycarbonate membranes (Corning; #3422). As for the invasion assays, the upper chamber had Matrigel (Corning; #354234) applied to it ahead of time and then it was left to set at 37°C for 30 min. Resuspend a total of 5 × 10^4^ cells per well in 200 µL of serum‐free media and plate on the top chamber. A total of 600 µL of the complete medium with 10% FBS as chemotaxicant was put into lower chamber and incubated for 24 h; then, the cells which did not migrate/invade on the upper surface of the filter membrane were taken off by cotton swab. Cells that traversed the membrane were fixed in 4% paraformaldehyde, stained with .1% crystal violet and imaged. Quantification was performed by enumerating cells in at least five randomly selected non‐edge fields per insert.

### 2.9 In vivo Western blot and co‐immunoprecipitation

Cells were extracted with RIPA buffer containing protease and phosphatase inhibitors. For co‐immunoprecipitation (Co‐IP), pre‐cleared lysates were incubated with the indicated antibodies or control IgG at 4°C overnight and subsequently pulled down using protein A/G magnetic beads (MCE; #HY‐K0202). Eluted immunoprecipitates were analysed by immunoblotting after separation by SDS‐PAGE and transfer to PVDF membranes.^28^ Sample blocked 5% NF milk. And then, it was put into the incubator for incubation with the primary antibodies at 4°C all night long and then treated with secondary antibody conjugated with HRP. For Co‐IPs using tagged constructs, cells were co‐transfected with FLAG–TMEM92 (or deletion mutants) and HA–DDX3X. Lysed cells were then incubated with anti‐FLAG antibodies for IP, and co‐precipitated DDX3X–HA was detected by Western blot with anti‐HA antibodies. TMEM92‐–MUT: TMEM92 deletion mutant without the L1 and L2 regions (ΔL1/ΔL2). The primary antibodies used for immunoblotting are listed below: antibodies against TMEM92, HER2, PR, ERα, DDX3X, TTC3, Flag, HA, His and β‐actin. Protein bands were detected using HRP‐conjugated secondary antibodies and chemiluminescence.

### 2.10 Immunohistochemistry and immunofluorescence

Paraffin‐embedded tumour tissues were sectioned, deparaffinized, rehydrated, and subjected to antigen retrieval. For immunohistochemistry (IHC), the prepared sections were incubated with the primary antibodies overnight. After this incubation time, they were incubated with a second antibody coupled to HRP and stained with 3,3′‐diaminobenzidine.^29^ Sections were sequentially probed with primary antibodies and Alexa Fluor‐labelled secondary antibodies (Invitrogen; #A32732), followed by DAPI nuclear staining. For immunofluorescence (IF) imaging, the stained cells were visualised, and high‐resolution images were captured using a Leica SP8 LIGHTNING super‐resolution confocal microscope (Leica Microsystems, Germany). IHC scoring was carried out using a semi‐quantitative approach according to the staining strength. TMEM92 immunoreactivity was independently evaluated by two blinded observers using a semi‐quantitative scale (0, negative; 1, weak; 2, moderate; 3, strong). For each specimen, the final IHC score was calculated as the average intensity across representative fields. The final IHC score was used in subsequent statistical analysis. IHC was performed with primary antibodies against TMEM92, DDX3X and Ki67.

### 2.11 Ubiquitination assay

Cells were cotransfected with HA‐DDX3X(1 µg/well), His‐Ubiquitin(1 µg/well) and other indicated plasmids for 48 h. MG132 (10 µM) was added for 6 h before lysing in a denaturing buffer. His‐tagged ubiquitinated protein was pulled down with nickel–nitrilotriacetic acid (Ni—NTA) agarose beads (Qiagen; #30210), and the ubiquitination of HA–DDX3X was analysed by Western blot. For ubiquitination assays, cells were transfected with HA‐DDX3X with Flag‐TTC3 and His‐Ubiquitin constructs (wild‐type [WT] or lysine mutants, K6O, K11O, K27O, K29O, K33O, K48O, K63O or K48R). Cells were added with MG132(10μ) for 6H prior to harvest. DDX3X ubiquitination was examined by anti‐HA immunoprecipitation coupled with anti‐His immunoblot analysis.

### 2.12 In vitro GST pull‐down and competitive binding assay

GST‐TMEM92, GST‐TTC3 and His‐DDX3X all were expressed and purified from *E. coli*. His‐tagged protein was purified with Ni‐NTA agarose columns and GST‐tagged protein was purified by glutathione–sepharose beads according to the instructions of the manufacturer. For pull‐downs, prey proteins were pulled down with the GST‐tagged protein that was immobilised on glutathione–sepharose beads (GE Healthcare; 17‐0756‐01). For binding assays to look at possible competition, we added a steady amount of GST‐TTC3 and His‐DDX3X to different amounts of TMEM92. We did Western blot on the bound proteins.

### 2.13 In vivo xenograft tumour model

Six‐week‐old female BALB/c nude mice were obtained from the Nanjing Model Animal Research Center. All animal experiments were approved by the Experimental Animal Ethics Committee of Huai'an First People's Hospital and conducted in accordance with institutional guidelines. To eliminate inter‐batch variability and ensure rigorous comparisons, all in vivo xenograft experiments were conducted using a strictly synchronised cohort under standardised conditions.

A total of 5 × 10^6^ MDA‐MB‐231 cells harbouring the indicated lentiviral constructs were subcutaneously injected into the right flank of each mouse. The mice were randomly assigned into specific groups (*n* = 5 per group). For the TMEM92 knockdown evaluation, mice were injected with cells stably expressing shNC, shTMEM92‐1 or shTMEM92‐2. For the in vivo rescue experiment, mice were injected with cells expressing shNC+Vector, shTMEM92+Vector or shTMEM92+DDX3X. For the chemotherapy sensitisation assay, mice bearing shNC or shTMEM92 tumours were further randomised to receive either the vehicle control or cisplatin (DDP).

Tumour dimensions and animal health were closely monitored. Tumour sizes were measured every 3–4 days using a digital vernier caliper, and tumour volumes were calculated using the formula: *V* = .5 × length × width^2^. For the chemotherapy treatment, therapeutic interventions were initiated when tumour volumes reached approximately 100 mm^3^. DDP (5 mg/kg) or an equal volume of control vehicle was administered via intraperitoneal injection twice a week. At the end of the experimental observation period, all mice were ethically euthanised. The subcutaneous tumours were surgically excised, accurately weighed, photographed and subsequently fixed in 4% paraformaldehyde for downstream histological and molecular analyses.

### 2.14 TUNEL assay

In situ cell death detection kit, fluorescein (Roche; #11684795910) was used to detect apoptosis in the xenograft tumour tissue according to the manufacturer's instructions. Paraffin‐embedded tumour sections were first deparaffinised and rehydrated. The sections were then permeabilised with Proteinase K (20 µg/mL) for 15 min at room temperature. Subsequently, the sections were incubated with the TUNEL reaction mixture in a humidified chamber at 37°C for 60 min. The reaction mixture included terminal deoxynucleotidyl transferase and fluorescein‐labelled dUTP. The incubation period passed and slides were washed with PBS. For cell nuclei to be seen, the sections were stained with DAPI. Once counterstained, the section were mounted with an anti‐fade medium. Then, we take the fluorescent image of the sections by fluorescence microscope (Leica DMi8). TUNEL‐positive (apoptotic) cells were quantified as a percentage of at least five random fields from each section.

### 2.15 Statistical analysis

Analytical processing was conducted using GraphPad Prism version 8. The presented quantitative outputs reflect the mean ± SD derived from no fewer than three independent assays. The selection between parametric and rank‐based algorithms depended on preliminary assumptions checking, utilising the Shapiro–Wilk protocol for distribution normality and Levene's test for variance uniformity. Consequently, inter‐group variances involving two conditions were probed via standard two‐tailed *t*‐tests or Mann–Whitney *U* evaluations. Conversely, variations across three or more categories were resolved through either one‐way ANOVA or the Kruskal–Wallis framework. For patient outcome modelling, we generated Kaplan–Meier prognostic curves and applied log‐rank testing to ascertain survival separations. The criterion for statistical significance was established at a two‐sided *p* value < .05.

## 3 RESULTS

### 3.1 Elevated TMEM92 predicts poor prognosis in TNBC

**FIGURE 1:**
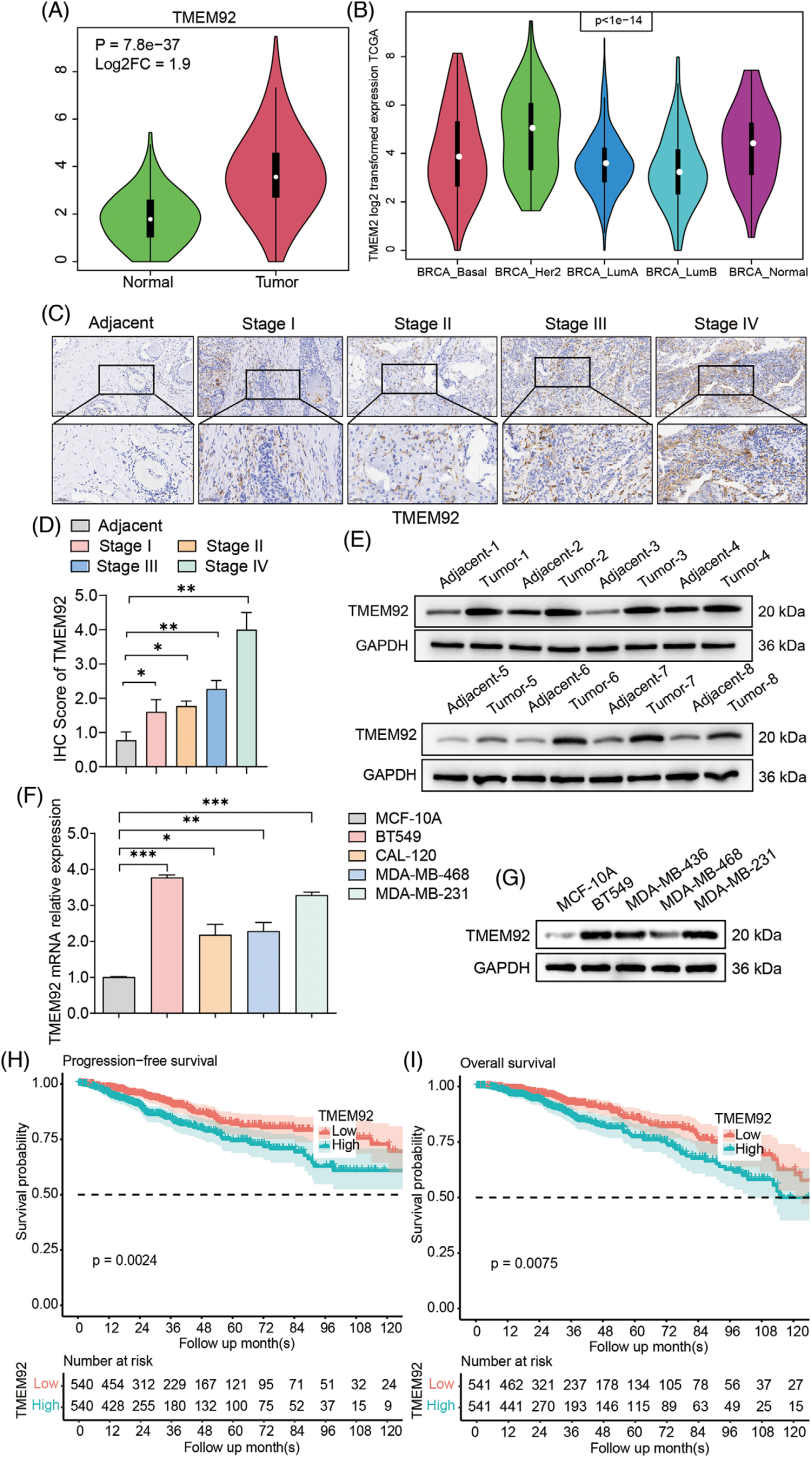
TMEM92 is elevated in triple‐negative breast cancer and predicts poor prognosis. (A) The mRNA expression of TMEM92 in breast cancer (BC) tissues and normal tissues from the TCGA database. (B) The mRNA expression of TMEM92 in different molecular subtypes of BC from the TCGA database. (C) Immunohistochemistry (IHC) staining of TMEM92 in breast cancer tissues of different pathological stages and adjacent normal tissues using a tissue microarray (TMA). Representative images are shown. Each group contains *n* = 80 independent patient samples. IHC staining was performed in three independent experimental batches. Scale bar: 100 µm. (D) IHC score analysis of TMEM92 expression levels in BC tissues of different clinical stages. (E) Western blot analysis of TMEM92 protein in matched BC and adjacent normal tissues (representative blots; *N* = 3 independent experiments). (F) The qRT‐PCR analysis of TMEM92 mRNA in different BC cell lines (mean ± SD, *N* = 3 biological replicates). (G) The basic TMEM92 protein level in different BC cell lines (representative blots; *N* = 3 independent experiments). (H and I) The Kaplan–Meier plot of progression‐free survival (PFS) and overall survival (OS) by the expression of TMEM92 in BC patients from the TCGA database. Statistical analysis: two‐tailed unpaired Student's *t*‐test or one‐way ANOVA as appropriate. Error bars represent mean ± SD. (*p < .05, **p < .01, ***p < .001).

TMEM92 expression in breast cancer tissues was evaluated using mRNA expression profiles obtained from The Cancer Genome Atlas (TCGA). TMEM92 expression was markedly elevated in tumour samples relative to matched adjacent normal tissues (log2FC = 1.9, *p* = 7.8 × 10^−^^3^ ^7^) (Figure 1A). Furthermore, molecular subtype‐stratified analysis showed that TMEM92 expression was elevated in basal‐like tumours, the molecular correlate of TNBC, and was also relatively high in the BRCA_Her2 subtype, compared with normal breast tissue (Figure 1B). Given that TNBC is defined by the absence of ER, PR and HER2 expression, we further examined whether TMEM92 knockdown affected the expression of these receptors. Western blot analysis showed that depletion of TMEM92 did not alter ER, PR or HER2 protein levels in TNBC cells, indicating that the biological effects of TMEM92 are independent of hormone receptor or HER2 status (Figure S2A). To corroborate these transcriptomic discoveries at the protein level, IHC staining demonstrated that the expression of TMEM92 in breast cancerous tissues was markedly higher compared with that in adjacent normal tissues. Moreover, TMEM92 expression showed an increasing trend across more advanced clinical stages (Figure 1C,D). However, this stage‐associated pattern should be interpreted with caution, as it may reflect a combination of intrinsic malignant biology and secondary clinical determinants, such as tumour burden and microenvironmental stress. Quantitative IHC scoring data for each clinical stage are summarised in Table S2. This was further corroborated by Western blot analysis of eight paired patient tissues, which confirmed a marked upregulation of TMEM92 protein in tumour versus adjacent normal tissues (Figure 1E). Moreover, we measured expression levels in different breast cancer cell lines and found significantly higher *TMEM92* mRNA and protein levels in TNBC cells (BT‐549, MDA‐MB‐468, MDA‐MB‐231) than in the non‐tumourigenic MCF‐10A epithelial cell line (Figure 1F,G). The relationship between the expression of TMEM92 and the prognosis of breast cancer patients was examined by utilising TCGA data. Patients with high TMEM92 expression had notably lower progression‐free survival (Figure 1H) and overall survival (Figure 1I) compared with those with low *TMEM92* expression (*p *= .0024 and *p* = .0075). To ensure the specificity of TMEM92 function, we performed CRISPR/Cas9‐mediated KO using two independent TMEM92‐KO clones and further conducted a rescue experiment by re‐expressing TMEM92 in KO cells. TMEM92 depletion markedly suppressed migration/invasion and clonogenic growth and increased cisplatin‐induced apoptosis, whereas TMEM92 re‐expression largely restored these phenotypes (Figures S2 and S3).

### 3.2 Knockdown of TMEM92 suppresses proliferation and migration of TNBC cells in vitro and in vivo

**FIGURE 2:**
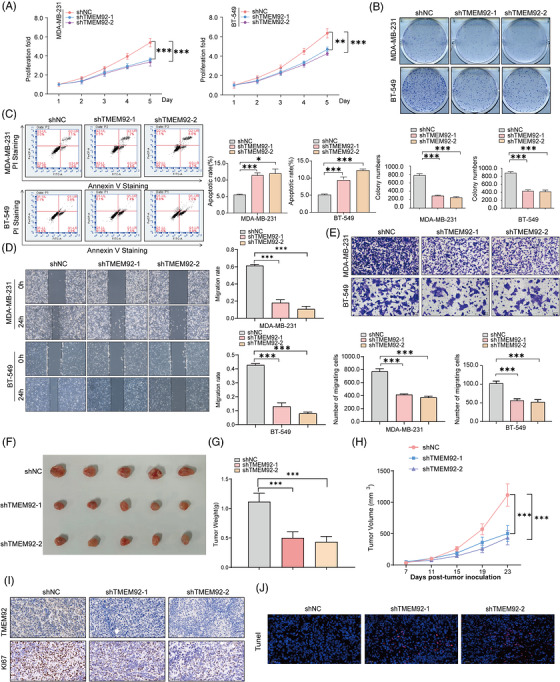
TMEM92 promotes the malignant phenotypes of TNBC cells. (A) Cell proliferation assay of control (shNC) and TMEM92‐knockdown (shTMEM92) TNBC cells (mean ± SD; *N* = 3 biological replicates). (B) Colony formation assay of control and shTMEM92 cells (*N* = 3 biological replicates). (C) Apoptosis assay of control and shTMEM92 cells by flow cytometry (representative plots; *N* = 3 independent experiments; quantification shown as % Annexin V^+^ cells, mean ± SD). (D) Wound healing assay of control and shTMEM92 cells (representative images; *N* = 3 independent experiments; wound closure quantified at indicated times, mean ± SD). (E) Transwell migration assay of control and shTMEM92 cells (representative images; *N* = 3 independent experiments; migrated cells quantified per field, mean ± SD). (F) Representative images of subcutaneous xenograft tumours formed from control and shTMEM92 cells. (G and H) Tumour weight and volume curves for xenograft tumours (mean ± SD). (I) IHC staining for TMEM92 and Ki67 in xenograft tumour tissues. (J) TUNEL staining for apoptosis in xenograft tumour tissues. Scale bars = 50 µm. Statistical analysis: two‐tailed unpaired Student's *t*‐test for two‐group comparisons; error bars represent mean ± SD; *N* values as indicated. (*p < .05, **p < .01, ***p < .001).

To investigate the functional role of TMEM92 in TNBC progression, we utilised two independent shRNAs to stably knockdown its expression in MDA‐MB‐231 and BT‐549 cells (Figure S3A). Findings from the cell proliferation experiments indicated that suppressing the expression of TMEM92 substantially impaired the viability of TNBC cells when contrasted with the negative control group, specifically the shNC group (Figure 2A). The findings from the colony formation tests revealed that the quantity of colonies formed by TMEM92‐silenced TNBC cells was notably diminished relative to control cells (Figure 2B). We performed flow cytometry to assess the effect of TMEM92 on apoptosis, and the results demonstrated that TMEM92 knockdown triggered a marked elevation in apoptotic rates in both cell lines. In BT‐549 cells, apoptosis increased by 4.52 and 7.15% in shTMEM92‐1 and shTMEM92‐2 groups, respectively; in MDA‐MB‐231 cells, the increases were 5.87 and 6.44% (*p* < .01) (Figure 2C). To evaluate the impact of TMEM92 on cell migration, wound healing and Transwell migration tests were carried out. In both tests, the migration of TNBC cells was notably reduced when TMEM92 was knocked down (Figure 2D,E). To validate the tumour‐enhancing function of TMEM92 in a living organism, we developed a subcutaneous xenograft model. In line with the outcomes obtained from in vitro experiments, tumours originating from TMEM92‐silenced cells demonstrated notably reduced growth rates. This led to smaller tumour sizes and lower ultimate tumour masses when compared with the control group (Figure 2F–H). Immunohistochemical analysis of xenograft tissues revealed reduced Ki67 staining in the shTMEM92 group, indicating decreased proliferation (Figure 2I). Furthermore, TUNEL assays confirmed a higher rate of apoptosis in the TMEM92‐knockdown tumours (Figure 2J). Taken together, these loss‐of‐function experiments demonstrate that TMEM92 is required for the proliferation, migration and survival of TNBC cells both in vitro and in vivo.

### 3.3 TMEM92 stabilises DDX3X by inhibiting its ubiquitination

**FIGURE 3:**
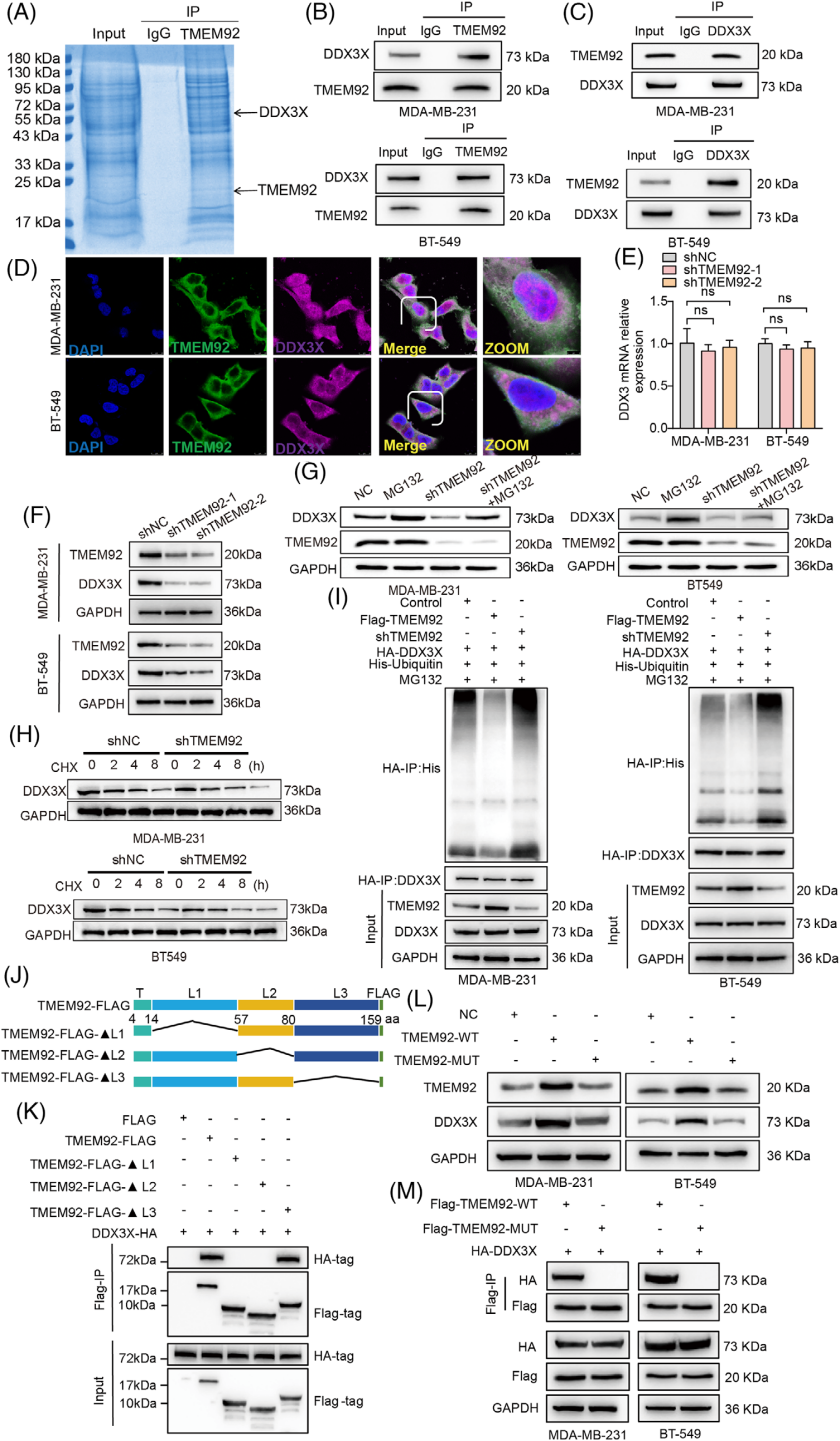
TMEM92 interacts with DDX3X and inhibits its proteasomal degradation. (A) Identification of TMEM92‐interacting proteins by Co‐IP and mass spectrometry (representative results; *N* = 3 independent Co‐IP/MS experiments). (B and C) Reciprocal endogenous Co‐IP of TMEM92 and DDX3X in TNBC cells (representative blots; *N* = 3 independent experiments). (D) Immunofluorescence showing partial co‐localisation of TMEM92 and DDX3X in the cytoplasm (representative images; *N* = 3 independent experiments). (E and F) The qRT‐PCR and Western blot analysis of DDX3X expression in control and shTMEM92 cells (qRT‐PCR: *N* = 3 biological replicates, mean ± SD; Western blot: representative blots; *N* = 3 independent experiments). (G) Western blot analysis of DDX3X in control and shTMEM92 cells with or without MG132 treatment (representative blots; *N* = 3 independent experiments). (H) CHX chase assay showing DDX3X protein stability in control and shTMEM92 cells (representative blots; *N* = 3 independent experiments). (I) In vivo ubiquitination assay showing the effect of TMEM92 on DDX3X ubiquitination (denaturing IP; representative blots; *N* = 3 independent experiments). (J) Schematic of TMEM92 deletion mutants. (K) Co‐IP assays using FLAG‐tagged full‐length and deletion mutants of TMEM92 to map the DDX3X‐interacting region. (L) Western blot analysis of DDX3X protein levels in cells expressing TMEM92‐WT or TMEM92‐MUT. (M) Co‐IP assays validating the interaction between DDX3X and TMEM92‐WT or TMEM92‐MUT (representative blots; *N* = 3 independent experiments). Statistical analysis (quantified assays): two‐tailed unpaired Student's *t*‐test; error bars represent mean ± SD; *N* values as indicated.

To elucidate the molecular mechanism by which TMEM92 promotes TNBC progression, we sought to identify its interacting protein partners. We performed Co‐IP of endogenous TMEM92 from MDA‐MB‐231 cell lysates, followed by mass spectrometry. This unbiased screen identified the DEAD‐box helicase DDX3X, a key regulator of mRNA translation and cell survival, as a high‐confidence binding partner of TMEM92 (Figure 3A). We validated this physical interaction by performing Co‐IP with an anti‐TMEM92 antibody, which successfully pulled down endogenous DDX3X in TNBC cells (Figure 3B). Reciprocally, Co‐IP using an anti‐DDX3X antibody also efficiently co‐precipitated endogenous TMEM92, confirming a robust interaction between the two proteins (Figure 3C). Furthermore, high‐resolution IF staining revealed striking spatial co‐localisation of TMEM92 and DDX3X, predominantly distributed within the cytoplasmic and perinuclear compartments of TNBC cells (Figure 3D). We next investigated the functional consequence of this interaction. TMEM92 depletion did not alter *DDX3X* mRNA expression levels (Figure 3E), it led to a marked reduction in DDX3X protein levels in both cell lines (Figure 3F). This suggested that TMEM92 might regulate DDX3X post‐translationally. In order to determine if this regulatory mechanism was associated with the ubiquitin–proteasome system, we administered the proteasome inhibitor MG132 to the cells. Notably, MG132 treatment completely rescued the downregulation of DDX3X protein induced by TMEM92 knockdown, indicating that TMEM92 protects DDX3X from proteasomal degradation (Figure 3G). To exclude the involvement of the alternative lysosomal degradation pathway, we treated cells with the autophagy inhibitor CQ and found it failed to rescue the downregulation of DDX3X protein in TMEM92‐deficient cells (Figure S4A). Consistent with this, CHX chase assays demonstrated that the protein half‐life of DDX3X was significantly shortened in TMEM92‐deficient cells compared with control cells (Figure 3H). To directly assess the role of TMEM92 in DDX3X ubiquitination, we performed an in vivo ubiquitination assay. This revealed that knockdown of TMEM92 substantially increased the polyubiquitination of DDX3X, a signal for proteasomal degradation (Figure 3I). Finally, to map the specific domain of TMEM92 required for its interaction with DDX3X, we constructed a series of FLAG‐tagged TMEM92 deletion mutants (Figure 3J). Co‐IP assays using these constructs revealed that deletion of the L1 and L2 domain (amino acids 14–80) completely abolished the interaction between TMEM92 and DDX3X, whereas other deletions did not affect binding (Figure 3K–M). Collectively, these findings uncover a novel regulatory mechanism where TMEM92, through its L1 and L2 domain, directly binds to DDX3X, thereby inhibiting its polyubiquitination and subsequent proteasomal degradation to maintain its protein stability.

### 3.4 TMEM92 exerts its oncogenic effects through DDX3X

**FIGURE 4:**
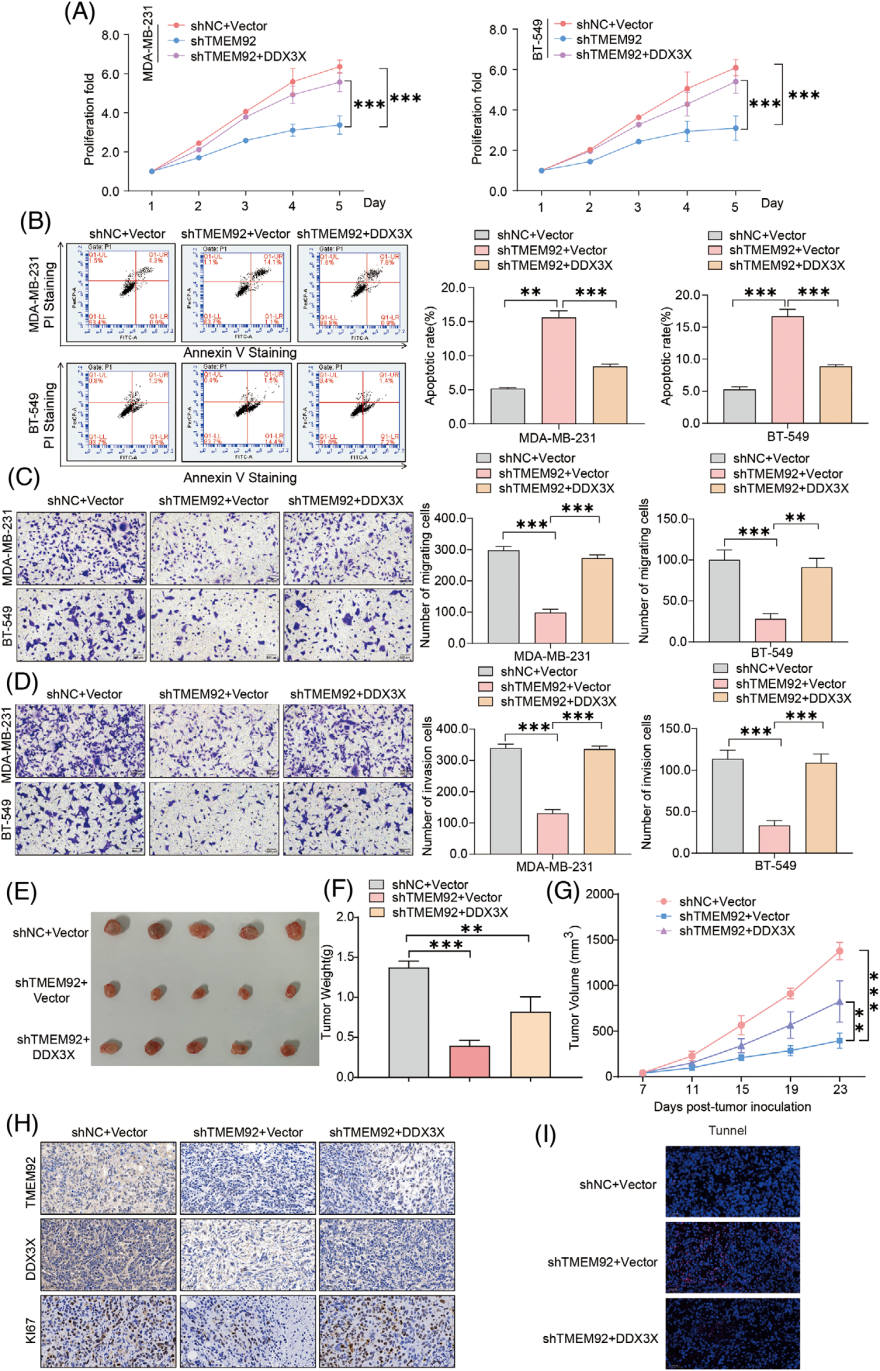
The pro‐tumourigenic effects of TMEM92 are mediated by DDX3X. (A) Cell proliferation assay of control (shNC+Vector), TMEM92‐knockdown (shTMEM92+Vector) and rescue (shTMEM92+DDX3X) cells (*N* = 3 biological replicates, mean ± SD). (B) Apoptosis assay of the indicated cell groups (representative plots; quantification shown as % Annexin V^+^ cells; *N* = 3 independent experiments, mean ± SD). (C and D) Transwell migration and invasion assays of the indicated cell groups (representative images; migrated/invaded cells quantified per field; *N* = 3 independent experiments, mean ± SD). (E) Representative images of subcutaneous xenograft tumours. (F and G) Tumour weight and volume curves for xenograft tumours from the indicated cell groups (mean ± SD). (H) IHC staining for TMEM92, DDX3X and Ki67 in xenograft tumour tissues. (I) TUNEL staining for apoptosis in xenograft tumour tissues. Scale bars = 50 µm. Statistical analysis: one‐way ANOVA with Tukey's post‐hoc test for multi‐group comparisons; error bars represent mean ± SD; *N* values as indicated. (**p < .01, ***p < .001).

To determine whether the pro‐tumourigenic effects of TMEM92 are mediated through its interaction with DDX3X, we performed a series of rescue experiments by overexpressing DDX3X in TMEM92‐knockdown TNBC cells. As expected, re‐introduction of DDX3X partially rescued the proliferation defects induced by TMEM92 depletion in both MDA‐MB‐231 and BT‐549 cells (Figure 4A). Similarly, overexpression of DDX3X significantly attenuated the increased apoptosis observed in TMEM92‐deficient cells (Figure 4B). We next examined whether DDX3X contributes to TMEM92‐dependent regulation of cell motility. Transwell migration and invasion assays showed that ectopic expression of DDX3X partially rescued the reduced migratory and invasive capacities caused by TMEM92 depletion (Figure 4C,D). Finally, we validated these findings in vivo using a subcutaneous xenograft model. Consistent with our in vitro data, the overexpression of DDX3X partially rescued the tumour growth inhibition caused by TMEM92 knockdown, as evidenced by restored tumour weight and volume (Figure 4E–G). Immunohistochemical analysis of the resected tumours confirmed that DDX3X re‐expression restored the levels of the proliferation marker Ki67 in TMEM92‐depleted tumours (Figure 4H). Furthermore, the increased apoptosis induced by TMEM92 knockdown was also reduced, as shown by TUNEL staining (Figure 4I). In summary, the pro‐tumourigenic functions of TMEM92 are critically dependent on its ability to stabilise DDX3X.

Together, these rescue experiments indicate that DDX3X acts as a critical downstream effector of TMEM92, while TMEM92 functions upstream to regulate TNBC progression by maintaining DDX3X stability.

### 3.5 TMEM92 stabilises DDX3X by competitively displacing the E3 ligase TTC3

**FIGURE 5:**
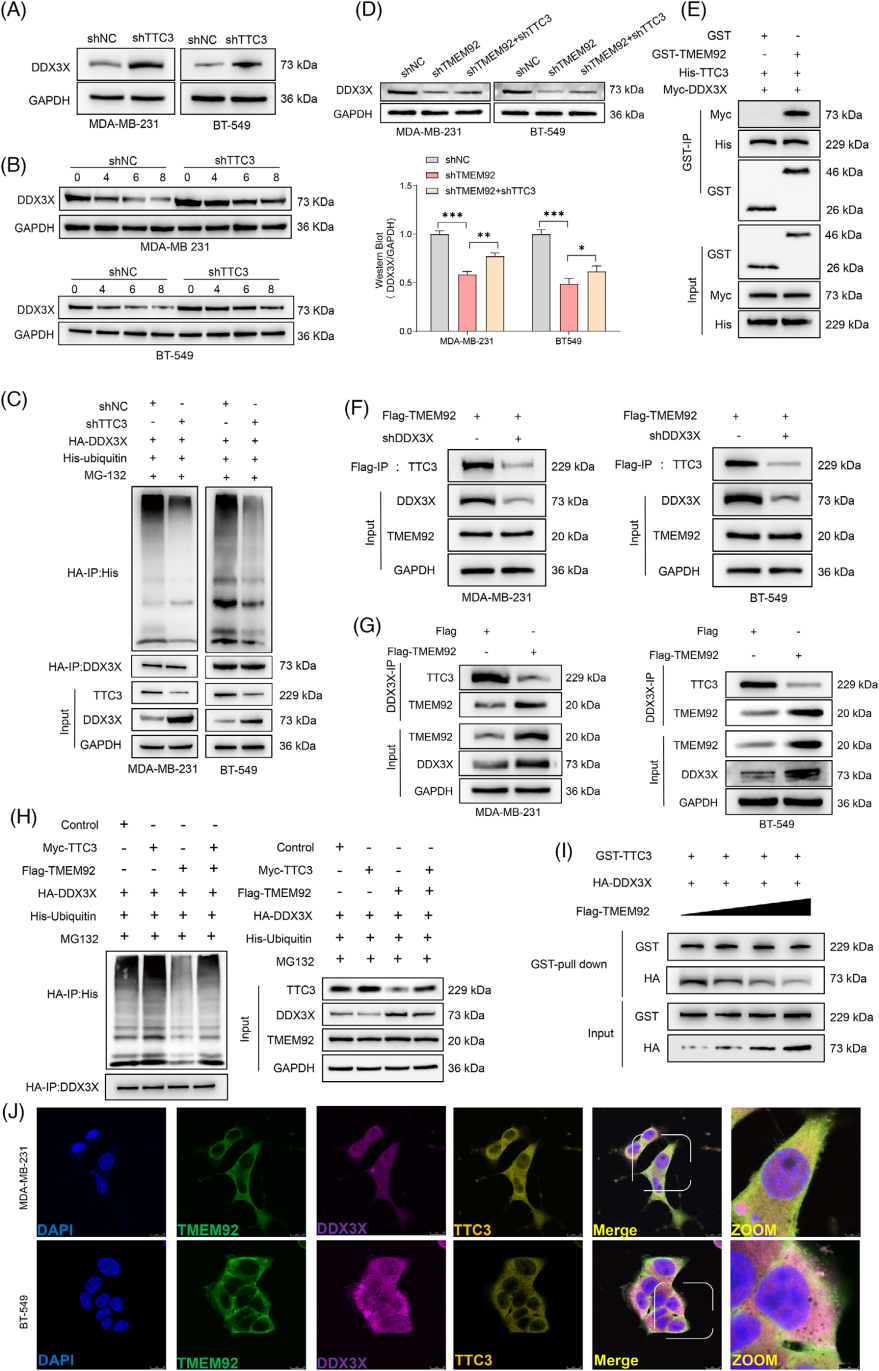
TMEM92 stabilises DDX3X by competitively inhibiting its ubiquitination by TTC3. (A) Western blot analysis of DDX3X in control and TTC3‐knockdown (shTTC3) cells (representative blots; *N* = 3 independent experiments;). (B) CHX chase assay of DDX3X stability in control and shTTC3 cells (representative blots; *N* = 3 independent experiments). (C) In vivo ubiquitination assay showing TTC3‐mediated ubiquitination of DDX3X (denaturing IP; representative blots; *N* = 3 independent experiments). (D) Western blot showing rescue of DDX3X levels by shTTC3 in TMEM92‐knockdown cells (representative blots; *N* = 3 independent experiments). (E) In vitro GST pull‐down assay showing direct interactions between TMEM92, TTC3 and DDX3X (representative gels; *N* = 3 independent pull‐downs). A weak background signal was observed in the GST‐only control, consistent with low‐level non‐specific binding commonly seen in GST pull‐down assays. (F and G) Co‐immunoprecipitation (Co‐IP) assays showing the DDX3X‐dependent interaction between TMEM92 and TTC3 in MDA‐MB‐231 and BT‐549 cells. In Figure 5G, cells transfected with Flag‐empty vector were used as a negative control for Flag‐TMEM92 overexpression (representative blots; *N* = 3 independent experiments). (H) In vivo competitive ubiquitination assay (denaturing IP under the indicated expression conditions; representative blots; *N* = 3 independent experiments). (I) In vitro binding assay showing that TMEM92 reduces TTC3 association with DDX3X (representative gels; *N* = 3 independent experiments). (J) Representative super‐resolution confocal images showing the spatial co‐localisation of TMEM92 (green), DDX3X (magenta) and TTC3 (yellow) in TNBC cells. Nuclei were counterstained with DAPI (blue). Scale bars = 25 µm. Enlarged views (ZOOM) highlight the overlapping signals in the perinuclear regions. Scale bars = 8 µm. Statistical analysis (for quantified datasets): two‐tailed unpaired Student's *t*‐test; error bars represent mean ± SD; *N* values as indicated.

To define the molecular mechanism underlying TMEM92‐mediated stabilisation of DDX3X, we sought to identify the responsible E3 ubiquitin ligase. Intriguingly, our initial mass spectrometry screen, which identified DDX3X, also revealed the E3 ligase TTC3 as a component of the TMEM92 protein complex. As an initial step, TTC3 was evaluated as a potential regulator, and its depletion resulted in a marked accumulation of DDX3X protein (Figure 5A). CHX chase assays further demonstrated that TTC3 depletion extended the protein half‐life of DDX3X (Figure 5B), and in vivo ubiquitination assays showed that TTC3 knockdown markedly reduced DDX3X polyubiquitination (Figure 5C). These results collectively establish TTC3 as a bona fide E3 ligase that targets DDX3X for proteasomal degradation. Critically, an epistasis experiment revealed that simultaneous knockdown of TTC3 partially restored the protein levels of DDX3X caused by TMEM92 depletion, indicating that TMEM92 functions by antagonising the E3 ligase activity of TTC3 (Figure 5D). To dissect the mechanism of this antagonism, in vitro GST pull‐down assays using purified proteins confirmed that both TMEM92 and TTC3 could directly bind to DDX3X (Figure 5E). In cells, Co‐IP assays revealed that all three proteins could form a ternary complex; however, this complex was dependent on DDX3X, as knockdown of DDX3X completely abolished the interaction between TMEM92 and TTC3, establishing DDX3X as a central scaffold (Figure 5F,G). This scaffold‐dependent architecture strongly suggested a competitive binding mechanism. Indeed, a cellular ubiquitination assay showed that TMEM92 overexpression potently inhibited the TTC3‐mediated polyubiquitination of DDX3X (Figure 5H). To assess the potential for direct physical competition, an in vitro binding assay using purified components was performed. Increasing amounts of TMEM92 were associated with a reduced interaction between TTC3 and DDX3X, supporting a competitive effect (Figure 5I). To further visualise the spatial relationship among these three proteins, high‐resolution triple IF staining was performed using confocal microscopy. Clear spatial overlap among TMEM92, DDX3X and TTC3 was observed, predominantly within the cytoplasmic and perinuclear compartments of TNBC cells, providing robust imaging‐based evidence for their functional interplay within a shared subcellular pool (Figure 5J). This competitive binding was also functionally relevant, as knockdown of TTC3 significantly rescued the impaired cell proliferation, migration and invasion phenotypes caused by TMEM92 depletion (Figure S5A–C). These findings delineate a mechanism where TMEM92 directly competes with the E3 ligase TTC3 for binding to DDX3X, thereby shielding it from ubiquitination and proteasomal degradation.

### 3.6 TTC3 mediates K48‐linked polyubiquitination of DDX3X

**FIGURE 6:**
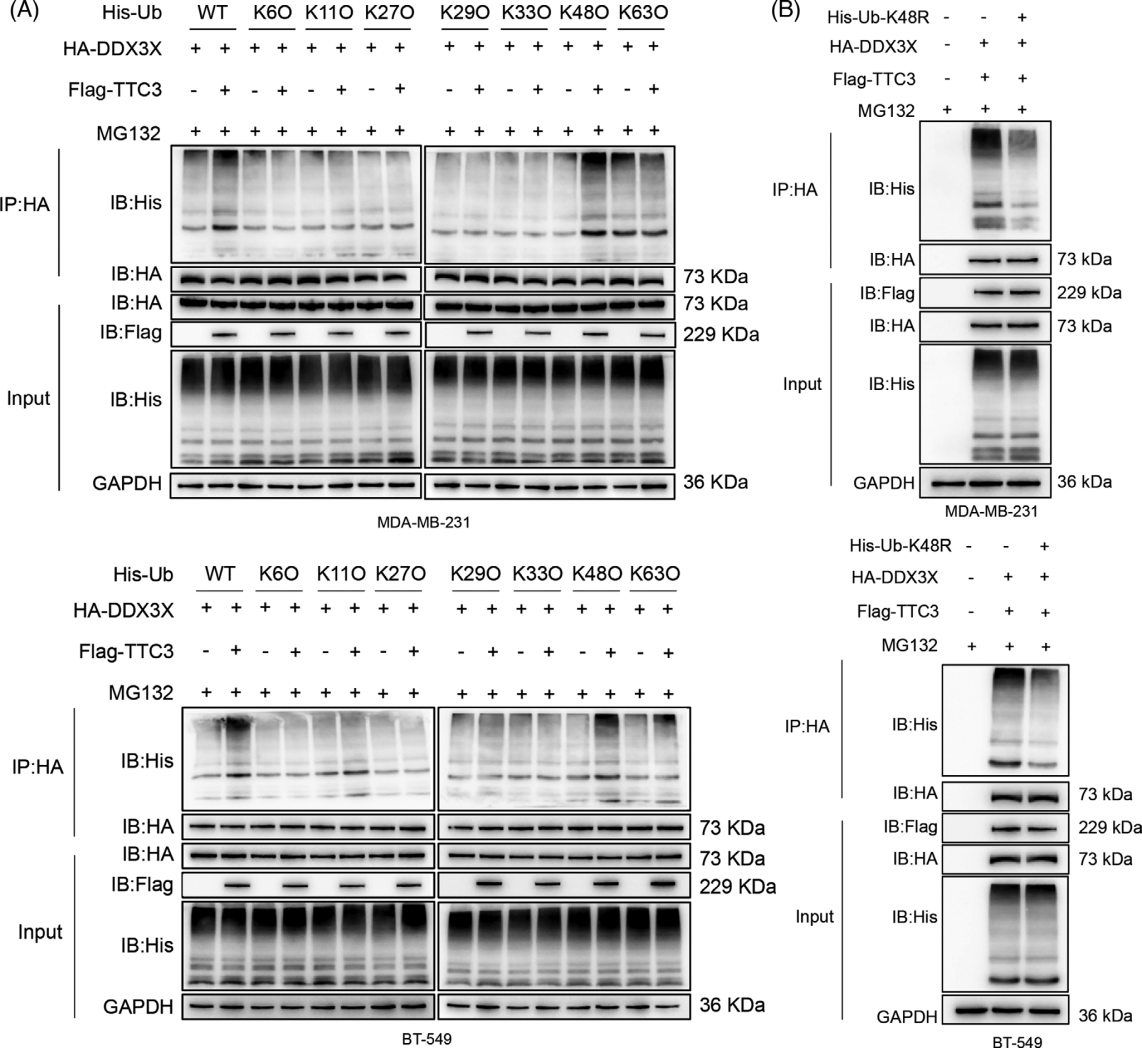
TTC3 promotes K48‐linked polyubiquitination of DDX3X. (A) In vivo ubiquitination assay using His‐tagged wild‐type (WT) and lysine‐only (K6O, K11O, K27O, K29O, K33O, K48O and K63O) ubiquitin mutants in MDA‐MB‐231 and BT‐549 cells (representative blots; *N* = 3 independent experiments). (B) In vivo ubiquitination assay using the His‐K48R ubiquitin mutant in MDA‐MB‐231 and BT‐549 cells to verify that TTC3‐induced polyubiquitination of DDX3X depends on K48‐linked ubiquitin chain formation (representative blots; *N* = 3 independent experiments).

To further delineate the specific ubiquitin linkage involved in TTC3‐mediated ubiquitination of DDX3X, we performed in vivo ubiquitination assays using a comprehensive panel of His‐tagged ubiquitin mutants in both MDA‐MB‐231 and BT‐549 cell lines. First, we utilised ‘K‐only’ ubiquitin mutants, in which all lysine residues except one were mutated to arginine, thereby allowing only one specific type of polyubiquitin chain formation. We observed that TTC3 overexpression markedly increase the polyubiquitination smear of DDX3X in the presence of WT ubiquitin. Notably, among the various linkage types, the promoting effect of TTC3 was most prominent when K48‐only (K48O) ubiquitin was employed, while its impact on other linkages, such as K63O, was significantly less pronounced (Figure 6A).

To further validate these findings, we utilised a K48R ubiquitin mutant, which specifically lacks the ability to form K48‐linked chains. In both cell lines, the robust polyubiquitination of DDX3X induced by TTC3 overexpression was attenuated in the K48R background (Figure 6B). Since K48‐linked polyubiquitination is the canonical signal that targets proteins for proteasomal degradation, these results collectively demonstrate that TMEM92 stabilises the DDX3X protein by specifically shielding it from TTC3‐mediated K48‐linked polyubiquitination, thereby preventing its subsequent degradation.

### 3.7 Knockdown of TMEM92 sensitises TNBC to DDP treatment

**FIGURE 7:**
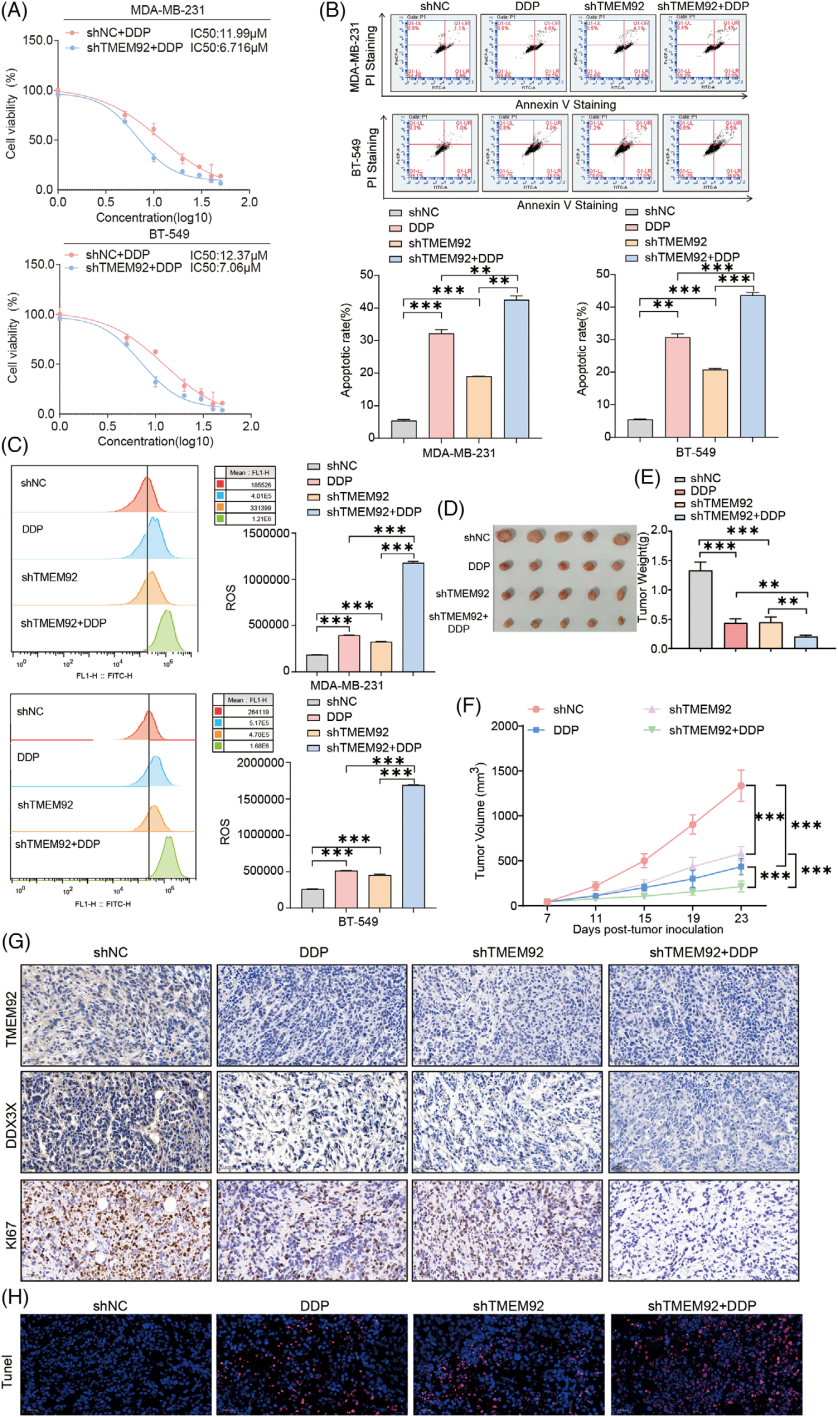
Knockdown of TMEM92 sensitises TNBC cells to DDP. (A) Cell viability assay of control and shTMEM92 cells treated with DDP (*N* = 3 biological replicates). (B) Apoptosis assay of the indicated cell groups after DDP treatment (representative flow plots; *N* = 3 independent experiments, quantification as % Annexin V^+^ cells, mean ± SD). (C) ROS level analysis in the indicated cell groups after DDP treatment (*N* = 3 independent experiments, mean ± SD). (D) Representative images of subcutaneous xenograft tumours from mice treated with DDP, shTMEM92 or combination therapy. (E and F) Tumour weight and volume curves for xenograft tumours. (G) IHC staining for TMEM92, DDX3X and Ki67 in xenograft tumour tissues. (H) TUNEL staining for apoptosis in xenograft tumour tissues. Scale bars = 50 µm. Statistical analysis: two‐tailed unpaired Student's t‐test or one‐way ANOVA as appropriate; error bars represent mean ± SD; *N* values as indicated. (*p < .05, **p <.01, ***p < .001).

The primary mechanism of DDP, a vital drug in the TNBC therapeutic arsenal, is the induction of overwhelming DNA damage leading to apoptosis.^30^ Since our data show the TMEM92–DDX3X axis is a key regulator of apoptosis, we reasoned that these two pathways converge. Therefore, we hypothesised that disrupting the TMEM92 axis would potentiate the cytotoxic effects of DDP. We first evaluated the effect of TMEM92 knockdown on the sensitivity of TNBC cells to DDP, a standard‐of‐care chemotherapeutic agent. Cell viability assays revealed that TMEM92‐deficient MDA‐MB‐231 and BT‐549 cells were significantly more sensitive to DDP, as evidenced by a substantial reduction in the IC50 values in the combination group compared with DDP treatment alone (Figure 7A). To understand the mechanism of this enhanced sensitivity, we assessed apoptosis. Flow cytometry analysis demonstrated that the combination of TMEM92 knockdown and DDP treatment induced a synergistic increase in apoptosis, far exceeding the levels observed with either treatment alone (Figure 7B). As DDP‐induced apoptosis is often mediated by reactive oxygen species (ROS), we measured ROS levels and found that while DDP or TMEM92 knockdown individually increased ROS, the combination treatment led to a significantly greater accumulation of ROS (Figure 7C). In addition to enhancing cell death, the combination of TMEM92 knockdown and DDP also resulted in a synergistic inhibition of cell invasion in Transwell assays (Figure S6A). Finally, to validate the therapeutic potential of targeting TMEM92 in vivo, we established a xenograft model and treated the mice with DDP, TMEM92 shRNA or a combination of both. While both individual treatments suppressed tumour growth compared with the control group, the combination of TMEM92 knockdown and DDP resulted in a profound and synergistic inhibition of tumour growth, leading to a dramatic reduction in final tumour weight and volume (Figure 7D–F). Immunohistochemical analysis of the excised tumours confirmed that the combination treatment led to the most significant decrease in TMEM92, DDX3X and the proliferation marker Ki67 (Figure 7G). Furthermore, TUNEL staining revealed a massive increase in apoptotic cells within the tumours from the combination treatment group, consistent with our in vitro findings (Figure 7H). Collectively, these results demonstrate that depletion of TMEM92 sensitises TNBC cells to DDP both in vitro and in vivo, suggesting that targeting the TMEM92–DDX3X axis is a promising strategy to enhance the efficacy of chemotherapy in TNBC. Clinically, platinum sensitivity in TNBC is often associated with homologous recombination deficiency (HRD) or BRCAness. To explore whether TMEM92 depletion sensitises TNBC cells by inducing an HRD‐like phenotype, we assessed cellular sensitivity to the PARP inhibitor olaparib. Notably, TMEM92 knockdown did not significantly alter olaparib sensitivity in either MDA‐MB‐231 or BT‐549 cells (Figure S7A), arguing against the induction of a classical HR‐deficient state sufficient to confer PARP inhibitor vulnerability. In contrast, IF analysis revealed that TMEM92 knockdown markedly increased DDP‐induced γ‐H2AX foci formation (Figure S7B), indicating aggravated DNA damage accumulation. These findings suggest that TMEM92 depletion enhances cisplatin sensitivity not by inducing canonical HR deficiency, but more likely by exacerbating chemotherapy‐induced damage and cellular stress.

### 3.8 TMEM92–DDX3X axis drives TNBC progression

**FIGURE 8:**
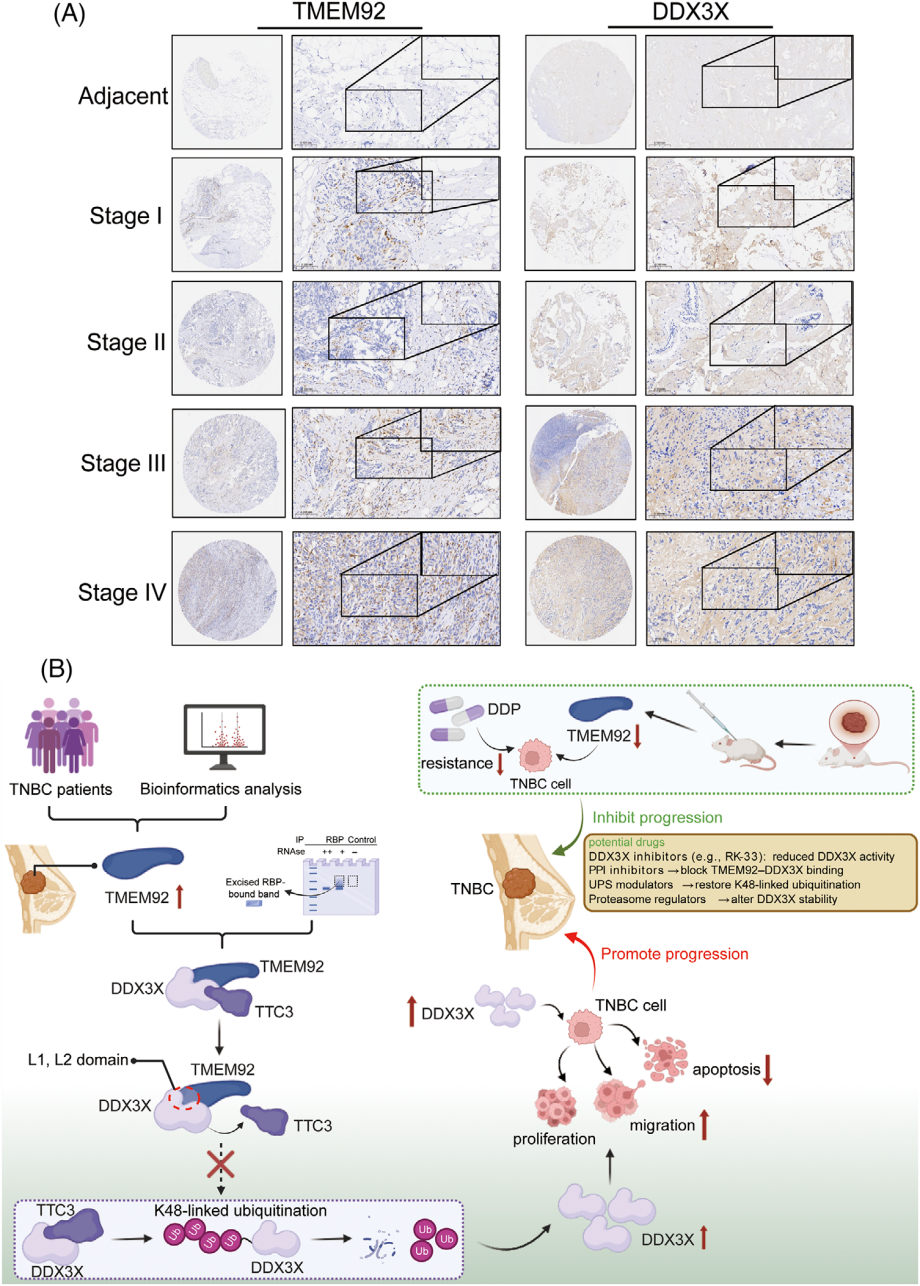
Co‐expression of TMEM92 and DDX3X in TNBC and proposed mechanism. (A) IHC staining of a TNBC tissue microarray shows a positive correlation between TMEM92 and DDX3X expression with tumour stage. (B) Model depicting how TMEM92 stabilises DDX3X by inhibiting TTC3‐mediated ubiquitination to promote TNBC progression and chemoresistance. Statistical analysis (for quantified TMA results): two‐tailed unpaired Student’ s *t*‐test; error bars represent mean ± SD.

Building on our findings regarding the role of TMEM92 in TNBC progression, we sought to investigate its clinical relevance and underlying mechanism. As shown by IHC staining of a TNBC tissue microarray, TMEM92 and its interacting partner DDX3X exhibit a positive correlation, and the expression levels of both proteins are significantly associated with a higher tumour stage, suggesting their co‐contribution to disease progression (Figure 8A). To further assess the clinical relevance of TMEM92 and DDX3X in TNBC, we analysed their associations with chemotherapy response using the ROC Plotter database. As shown in (Figure S8A), TMEM92 expression was relatively higher in non‐responders than in responders, and ROC analysis indicated a significant association between TMEM92 expression and pathological response to chemotherapy (AUC = .587, *p* = 4.6 × 10^−4^). DDX3X expression was also significantly associated with chemotherapy response (AUC = .561, *p* = 1.3 × 10^−2^), although its discriminative performance was modest (Figure S8B). Together, these data provide supportive clinical evidence linking TMEM92/DDX3X expression to chemotherapy non‐response in TNBC. This clinical observation is consistent with our in vitro and in vivo results, which demonstrate that the pro‐tumourigenic effects of TMEM92 are critically dependent on its ability to stabilise DDX3X. Mechanistically, we propose that TMEM92 functions by inhibiting TTC3‐mediated ubiquitination of DDX3X, thereby preventing DDX3X degradation and promoting TNBC cell proliferation, invasion and chemoresistance (Figure 8B). These findings establish a novel TMEM92–DDX3X signalling axis that drives TNBC progression and chemoresistance, providing a strong rationale for targeting this pathway as a potential therapeutic strategy.

## 4 DISCUSSION

TNBC persists as the most virulent subtype of breast carcinoma. It is distinguished by elevated frequencies of recurrence and metastasis, along with a glaring absence of targeted treatment options.^31^, ^32^, ^33^ This clinical reality drives the urgent need to identify novel molecular vulnerabilities that can be exploited for therapeutic intervention.^34^, ^35^, ^36^ In this study, we identify TMEM92 as a critical oncogenic driver in TNBC. We demonstrate that TMEM92 is highly expressed in TNBC, where it is associated with poorer survival outcomes. Mechanistically, we uncover a novel regulatory axis wherein TMEM92 promotes TNBC progression by directly interacting with the RNA helicase DDX3X, shielding TTC3‐mediated K48‐linked ubiquitination and proteasomal degradation. Crucially, we show that targeting this axis profoundly sensitises TNBC cells to DDP, a standard‐of‐care chemotherapy. These findings collectively establish the TMEM92–DDX3X axis as a central node in TNBC pathogenesis and a promising target for future therapies.

Our initial findings established TMEM92 not merely as a prognostic marker but as a bona fide oncogenic driver in TNBC. Through comprehensive loss‐of‐function studies, we demonstrated that depletion of TMEM92 markedly suppressed cell proliferation and migration while simultaneously inducing significant apoptosis in vitro and in vivo. While other members of the TMEM protein family have been implicated in various aspects of cancer biology, such as cell signalling and adhesion, a specific functional role for TMEM92 in solid tumours, particularly TNBC, has remained poorly characterised.^31^, ^37^, ^38^ Our work is the first to delineate a critical role for TMEM92 in sustaining the malignant phenotypes that define the aggressive nature of TNBC, thereby supporting its potential as a therapeutic target. TMEM92 expression was lower in MCF‐10A cells (∼30.4% of MDA‐MB‐231), suggesting tumour‐preferential expression and a potentially favourable therapeutic window. However, we acknowledge that a comprehensive evaluation of on‐target toxicity and off‐target effects will require systematic expression profiling across normal tissues and formal toxicological assessments in vivo. Such analyses are beyond the scope of the current mechanistic study and will depend on the future development of specific TMEM92‐targeting agents.

A central finding of our study is the elucidation of the novel TMEM92–DDX3X–TTC3 regulatory axis. We have delineated a precise molecular mechanism where TMEM92 ensures the stability of DDX3X by acting as a competitive inhibitor of its E3 ligase, TTC3. Importantly, this regulation selectively targets the canonical K48‐linked polyubiquitination pathway, providing a direct mechanistic link between competitive E3 exclusion and proteasome‐dependent protein stability. This is achieved through a DDX3X‐scaffolded ternary complex, where TMEM92 physically competes with TTC3 for access to DDX3X, thereby obstructing its ubiquitination. The role of DDX3X in oncology has been controversially described as paradoxical, with reports suggesting both tumour‐suppressive and oncogenic functions depending on the cancer type.^24^, ^39^, ^40^ For example, reduced DDX3 expression has been observed in advanced colorectal cancer and is associated with poor prognosis, supporting a tumour‐suppressive role.^39^ In contrast, in TNBC, DDX3X is frequently overexpressed, and pharmacological inhibition of DDX3X (e.g., RK33) synergises with platinum‐based chemotherapy, indicating a predominantly oncogenic function.^24^ Moreover, under hypoxic and nutrient‐deprived conditions, DDX3X can undergo proteolytic cleavage and promote tumour progression through aberrant RNA splicing regulation, highlighting its functional plasticity.^40^ Our data, however, unequivocally demonstrate that in the context of TNBC, the pro‐tumourigenic functions of DDX3X are dominant and are critically dependent on the stabilising effect of TMEM92. Whether TMEM92 binding also influences the RNA‐binding or helicase activity of DDX3X remains to be determined and warrants further investigation. To our knowledge, the regulation of an RNA helicase's stability through the competitive displacement of its E3 ligase by a TMEM protein represents a previously undescribed paradigm in protein stability regulation, which deepens our understanding of TNBC biology. Interestingly, while canonical annotations often predict TMEM family proteins to localise at the plasma membrane or nucleus, our in vitro high‐resolution IF data reveal a substantial pool of the TMEM92–DDX3X–TTC3 complex enriched in the cytoplasmic and perinuclear regions of TNBC cells. Furthermore, our in vivo IHC staining revealed TMEM92 expression in both the nucleus and cytoplasm. As suggested by recent studies, DDX3X is a dynamic RNA helicase capable of nucleocytoplasmic shuttling.^41^, ^42^ It is highly plausible that dynamic shuttling and context‐dependent re‐localisation occur in aggressive TNBC cells, allowing TMEM92 and DDX3X to share a functional spatiotemporal pool. We hypothesise that this partial cytoplasmic/perinuclear overlap provides the necessary spatial basis for TMEM92 to interact with DDX3X and shield it from TTC3‐mediated degradation.

A major hurdle in TNBC treatment is the frequent development of intrinsic or acquired resistance to platinum‐based chemotherapy.^43^, ^44^, ^45^ Our study provides a compelling rationale for targeting TMEM92 as a chemosensitisation strategy. We demonstrated that knockdown of TMEM92 profoundly sensitised TNBC cells to DDP‐induced apoptosis, an effect linked to the exacerbation of ROS. While established mechanisms of DDP resistance often involve enhanced DNA repair pathways^46^ or altered ROS metabolism,^47^, ^48^ our findings introduce the TMEM92–DDX3X axis as a novel, complementary pathway governing chemo‐sensitivity. This opens exciting therapeutic avenues, such as the development of TMEM92‐targeting agents – including small molecules, antibodies or PROTACs – to be used in combination with standard chemotherapy to overcome resistance and improve patient outcomes.

Clinically, platinum efficacy in TNBC is frequently linked to HRD or a BRCAness phenotype. However, our supporting information suggest that TMEM92‐mediated chemoresistance is unlikely to operate primarily through canonical HR pathways. Specifically, TMEM92 depletion did not sensitise TNBC cells to PARP inhibition, arguing against the induction of a classical HR‐deficient state sufficient to confer therapeutic vulnerability. Instead, TMEM92 knockdown markedly increased DDP‐induced γ‐H2AX foci, indicating aggravated DNA damage accumulation. Taken together with the observed exacerbation of ROS, the current evidence favours a model in which TMEM92 loss lowers cellular tolerance to chemotherapy‐induced stress rather than acting as a canonical HR regulator. From a translational perspective, this HR‐independent mechanism is highly encouraging, as it raises the possibility that TMEM92 targeting may hold therapeutic relevance beyond HR‐deficient populations, potentially extending the benefit of platinum‐based therapies to HR‐proficient TNBC patients.

We acknowledge several limitations in our study. First, while our prognostic analyses are compelling, they are primarily based on public databases and require validation in larger, multi‐centre clinical cohorts to firmly establish TMEM92 as an independent prognostic biomarker. While our data indicate a strong association between TMEM92 expression and adverse patient survival (both PFS and OS), these findings from large‐scale public datasets should be interpreted with caution. As in any retrospective big‐data survival analysis, overall survival is a composite metric inherently susceptible to confounding variables, including treatment heterogeneity and post‐progression interventions. Therefore, the observed survival association cannot be exclusively attributed to direct chemotherapy resistance. Biologically, it is highly plausible that TMEM92 confers broader stress‐adaptive functions – such as enhancing cellular tolerance to oxidative stress (e.g., ROS) or metabolic pressures – which may help tumour cells survive subsequent lines of therapy or influence post‐progression survival. Furthermore, we explicitly acknowledge that retrospective analyses may overestimate the independent contribution of any single molecular factor to overall survival. Similarly, while we observed a positive correlation between TMEM92 expression and advanced clinical stages (Figure 1C,D), it is important to note that clinical staging is a multifactorial metric heavily influenced by tumour burden, biological subtype and treatment history. Therefore, the observed stage‐associated increase in TMEM92 expression may reflect a combination of underlying biological processes – such as an increased proliferative index in larger tumours or hypoxic adaptation within the poorly vascularised core of advanced masses – rather than acting solely as a direct, independent marker of tumour aggressiveness. Future studies rigorously controlling for molecular subtypes, treatment history and independent proliferative markers (e.g., Ki‐67) are warranted to dissect whether TMEM92 is a primary driver of malignant progression or a secondary stress‐response marker associated with advanced tumour volume. In addition, our work mainly focused on the cell‐autonomous role of TMEM92 within TNBC cells. Whether TMEM92 may also exert systemic, non‐cell‐autonomous effects remains an open and intriguing question. For example, TMEM92 overexpression in specific organs such as the liver could potentially influence the secretion of circulating factors that create a microenvironment permissive for tumour growth and metastasis. Future studies employing approaches such as AAV‐mediated tissue‐specific overexpression will be necessary to explore this possibility and more comprehensively define the systemic roles of TMEM92 in cancer biology.

Second, our mechanistic investigation focused exclusively on the TMEM92–DDX3X–TTC3 axis. It remains possible that TMEM92 has other binding partners or that DDX3X stabilisation influences downstream pathways beyond those explored here. While the present study focuses on the proximal regulation of DDX3X stability by TMEM92, future studies exploring the transcriptional or signalling mechanisms governing TMEM92 expression may further expand this regulatory network. Moreover, although increased ROS accumulation was observed following TMEM92 knockdown combined with cisplatin treatment, ROS scavenger–based rescue experiments (e.g., NAC) were not performed. Therefore, the causal contribution of ROS to the observed chemosensitisation warrants further validation in future studies. Future research should therefore aim to broaden the TMEM92 interactome and downstream signalling network. Finally, our study is preclinical; the definitive therapeutic potential of targeting TMEM92 requires the development of specific pharmacological inhibitors and their subsequent testing in more clinically relevant models, such as patient‐derived xenografts (PDXs), before consideration for clinical trials. Furthermore, we acknowledge that while our preclinical in vitro and in vivo models robustly demonstrate the role of the TMEM92–DDX3X axis in mediating cisplatin resistance, direct clinical validation specifically for platinum‐based therapy remains a limitation of the current study. Our exploratory analysis of large‐scale transcriptomic datasets (e.g., ROC Plotter) confirms that TMEM92 and DDX3X expression significantly predicts poor response to general neoadjuvant chemotherapy in TNBC patients (Figure S8). However, due to the scarcity of well‐annotated public cohorts treated strictly with platinum monotherapy, providing definitive clinical proof of platinum‐specific resistance is challenging at the mRNA level. Moreover, since TMEM92 regulates DDX3X stability primarily via post‐translational ubiquitination rather than transcriptional control, mRNA abundance may not fully reflect the functional protein status in patients. Therefore, our conclusions regarding clinical chemoresistance should be interpreted with caution. Future validation in larger, well‐annotated clinical cohorts – particularly those utilising quantitative proteomics or IHC on paired pre‐ and post‐treatment biopsies – is required to firmly establish the translational value of this axis as a platinum‐specific biomarker.

To sum up, our research pinpoints TMEM92 as a crucial oncogenic factor in TNBC. It fosters tumour advancement by stabilising DDX3X via a novel competitive binding process. This mechanism impedes the degradation of DDX3X by the E3 ligase TTC3. Our work reveals the TMEM92–DDX3X axis as a significant therapeutic vulnerability in TNBC. Moreover, it validates this axis as a potentially effective therapeutic target, especially as an approach to surmount the widespread problem of chemoresistance.

Beyond cisplatin‐based chemotherapy, the TMEM92–DDX3X–TTC3 signalling axis presents multiple potential pharmacological intervention points in TNBC. First, direct inhibition of DDX3X represents a rational strategy, as DDX3X functions as a key downstream effector of this axis. Small‐molecule DDX3X inhibitors, such as RK‐33, have been reported to suppress tumour growth and sensitise cancer cells to chemotherapy and may therefore attenuate the oncogenic output driven by TMEM92‐mediated DDX3X stabilisation. Second, given that DDX3X stability is regulated by TTC3‐mediated K48‐linked ubiquitination, pharmacological modulation of the ubiquitin–proteasome system may indirectly influence this pathway by restoring DDX3X turnover. Third, disruption of the protein–protein interaction between TMEM92 and DDX3X could, in principle, relieve the inhibitory effect of TMEM92 on TTC3‐dependent ubiquitination, thereby limiting aberrant DDX3X accumulation. Finally, as DDX3X plays a central role in RNA metabolism and translational regulation, agents targeting translational control or RNA processing may indirectly suppress the tumour‐promoting consequences of TMEM92–DDX3X–TTC3 axis activation. Collectively, these conceptual therapeutic strategies highlight the druggability of this signalling axis and provide a framework for developing combination therapies to overcome therapeutic resistance in TNBC.

## AUTHOR CONTRIBUTIONS

Hao Shen, Xiaochao Jia and Xu Li contributed equally to this work. Hao Shen, Xiaochao Jia and Xu Li designed and performed most of the experiments, analysed the data and drafted the manuscript. Zhi Li performed additional experiments during manuscript revision and organised the corresponding data. Zhihua Zhang and Yang Zhao assisted with in vitro and in vivo experiments. Lei Shen, Xiaoqiu Bu, Qiang Ma and Chunli Liang provided technical support and helped with data collection. Xiaoti Lin, Lin‐Xiaoxi Ma and Chuan Qin conceived and supervised the project, revised the manuscript critically for important intellectual content and provided overall guidance. All authors discussed the results and approved the final version of the manuscript.

## CONFLICT OF INTEREST STATEMENT

The authors declare no conflicts of interest.

## ETHICS STATEMENT

The animal experiments conducted in this study were reviewed and approved by the Experimental Animal Ethics Committee of Huai'an First People's Hospital (Approval No. DW‐P‐2024‐034‐01). All methods were carried out in accordance with the institutional guidelines for the care and use of laboratory animals.

## Supporting information



Supporting Information

Supporting Information

Supporting Information

Supporting Information

Supporting Information

Supporting Information

Supporting Information

Supporting Information

Supporting Information

Supporting Information

Supporting Information

## Data Availability

Data will be made available on request.
