# Supplementary figures and images for: TMEM92 shields DDX3X from TTC3‐mediated degradation to confer chemoresistance in triple‐negative breast cancer
Source: Clin Transl Med. 2026 May 15;16(5):e70681. doi: 10.1002/ctm2.70681 (PMC13178151; doi:10.1002/ctm2.70681)

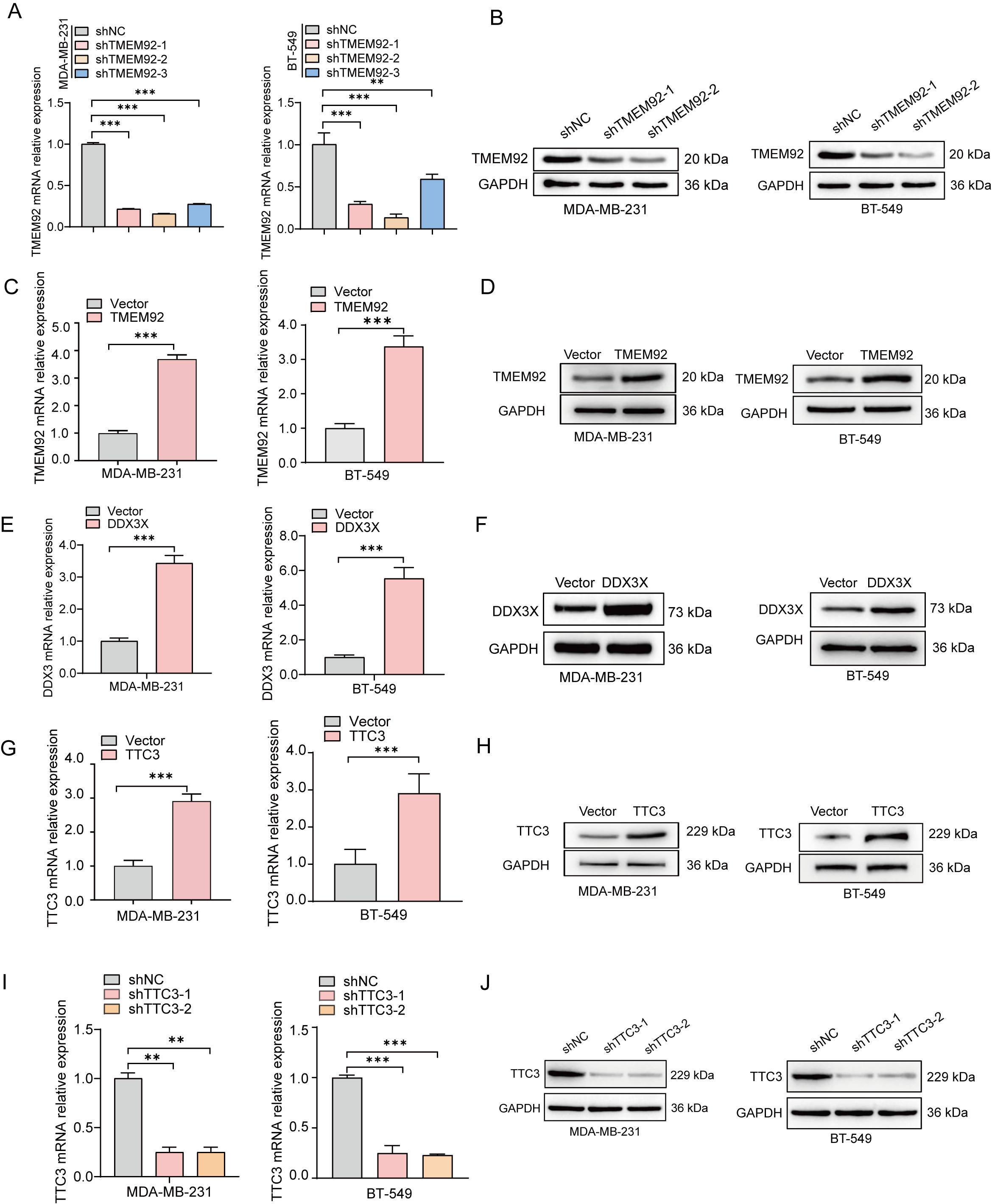

Supplement: Supplementary file 1 — Supporting Information [file CTM2-16-e70681-s006.tif]

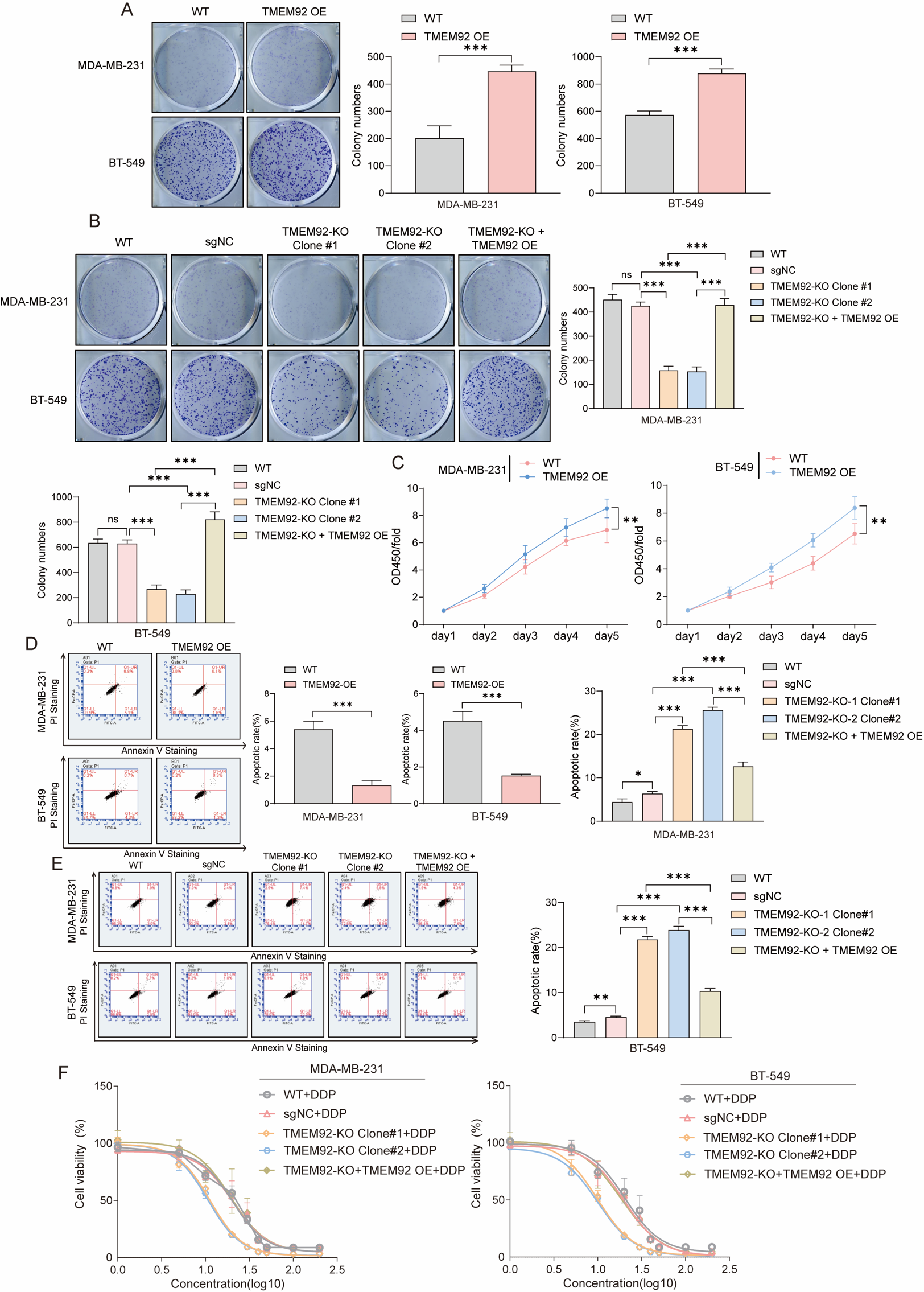

Supplement: Supplementary file 3 — Supporting Information [file CTM2-16-e70681-s002.pdf]

A

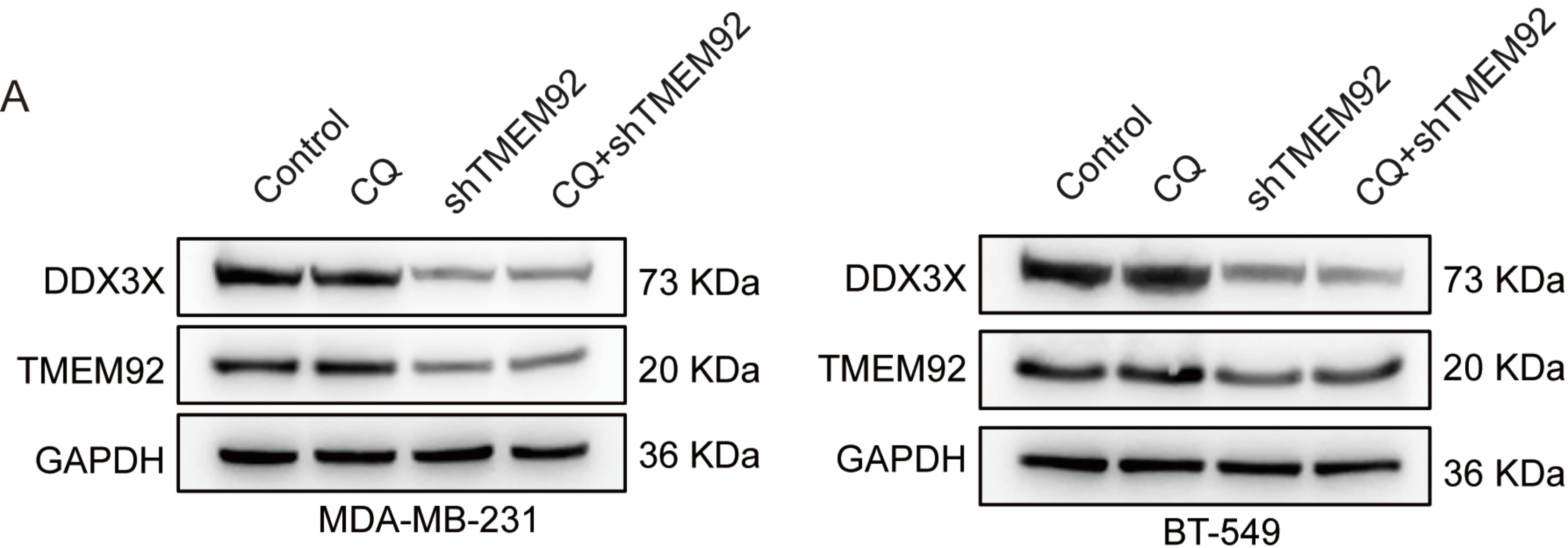

Supplement: Supplementary file 4 — Supporting Information [file CTM2-16-e70681-s011.pdf]

A

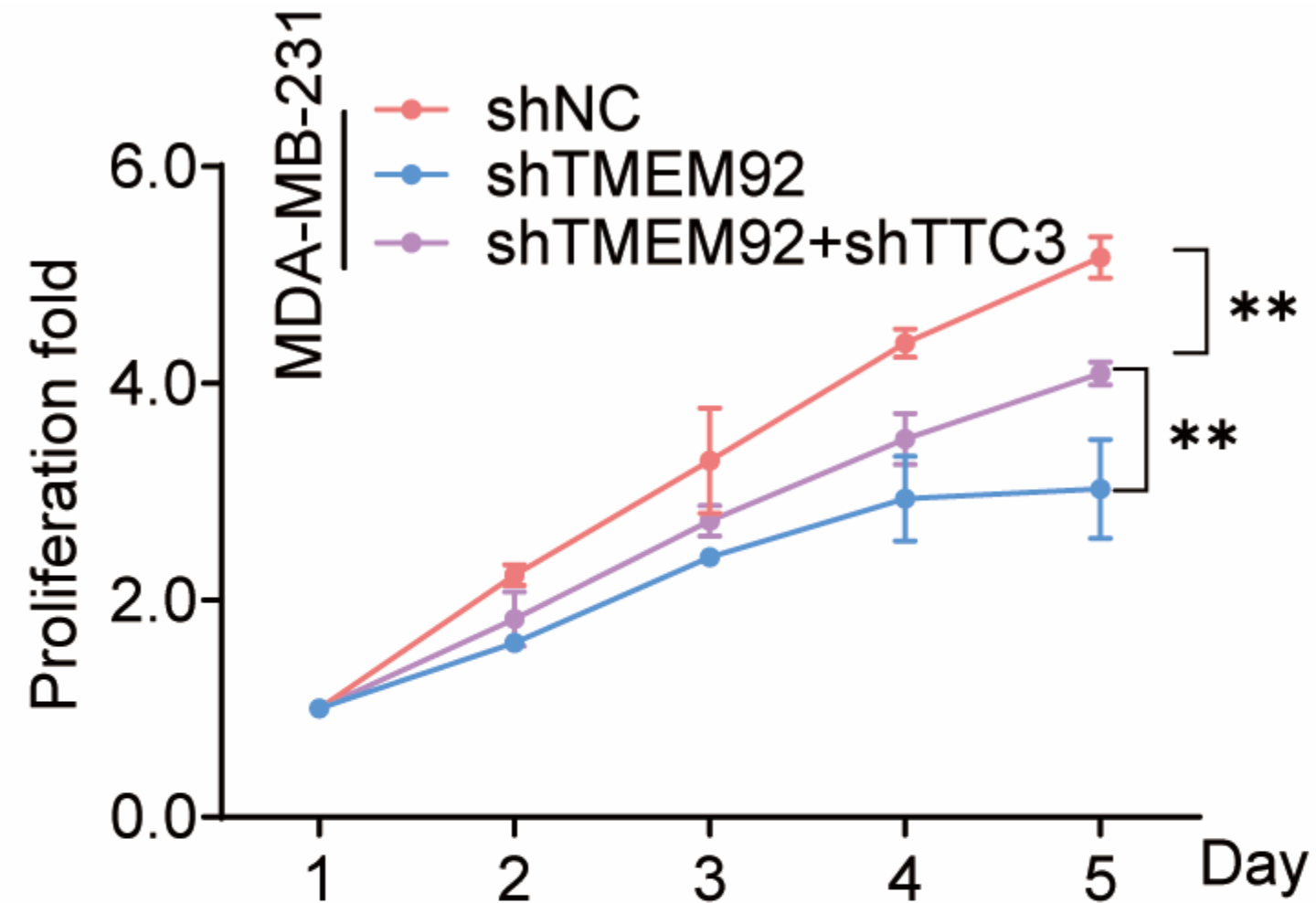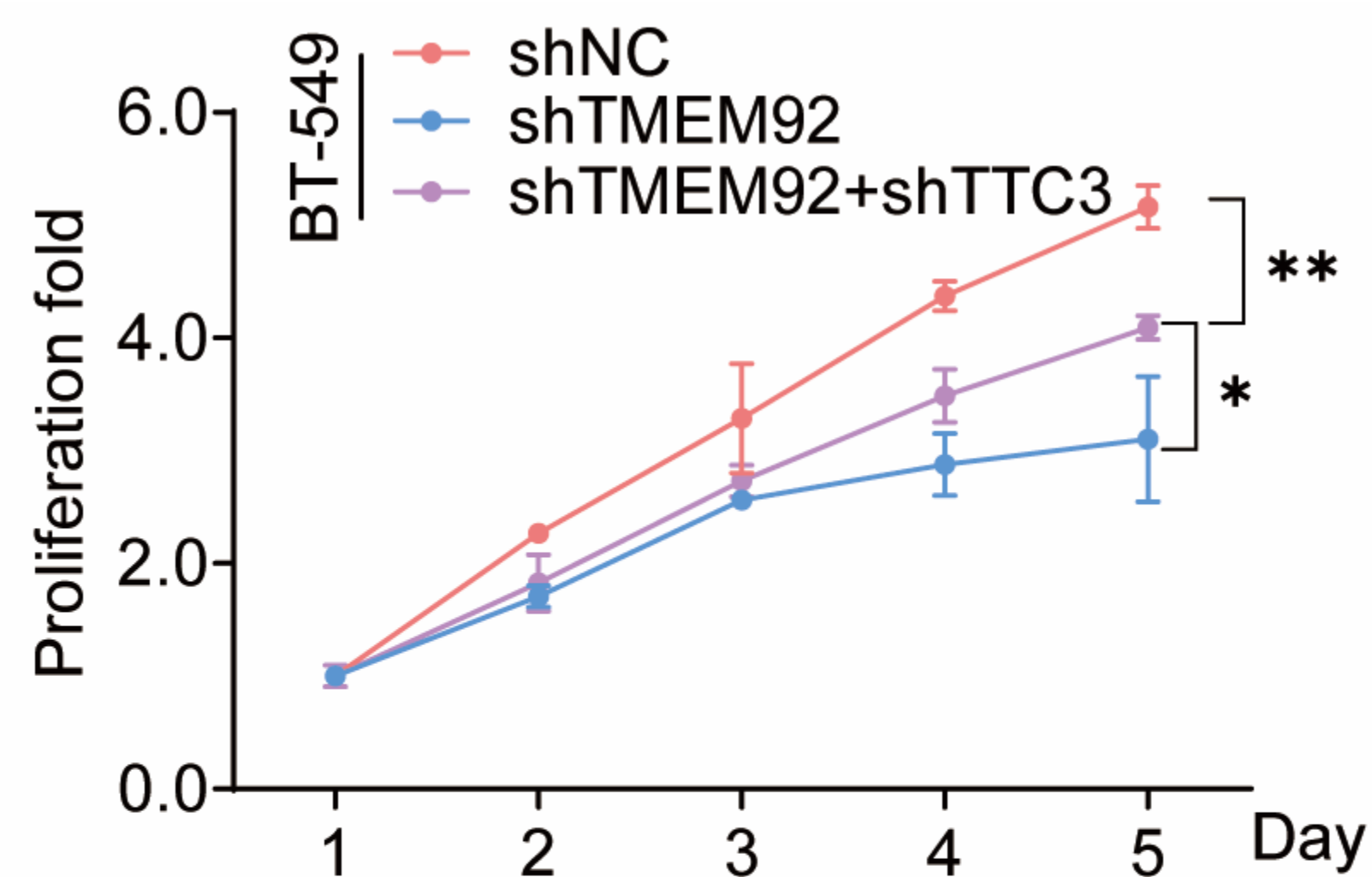

B

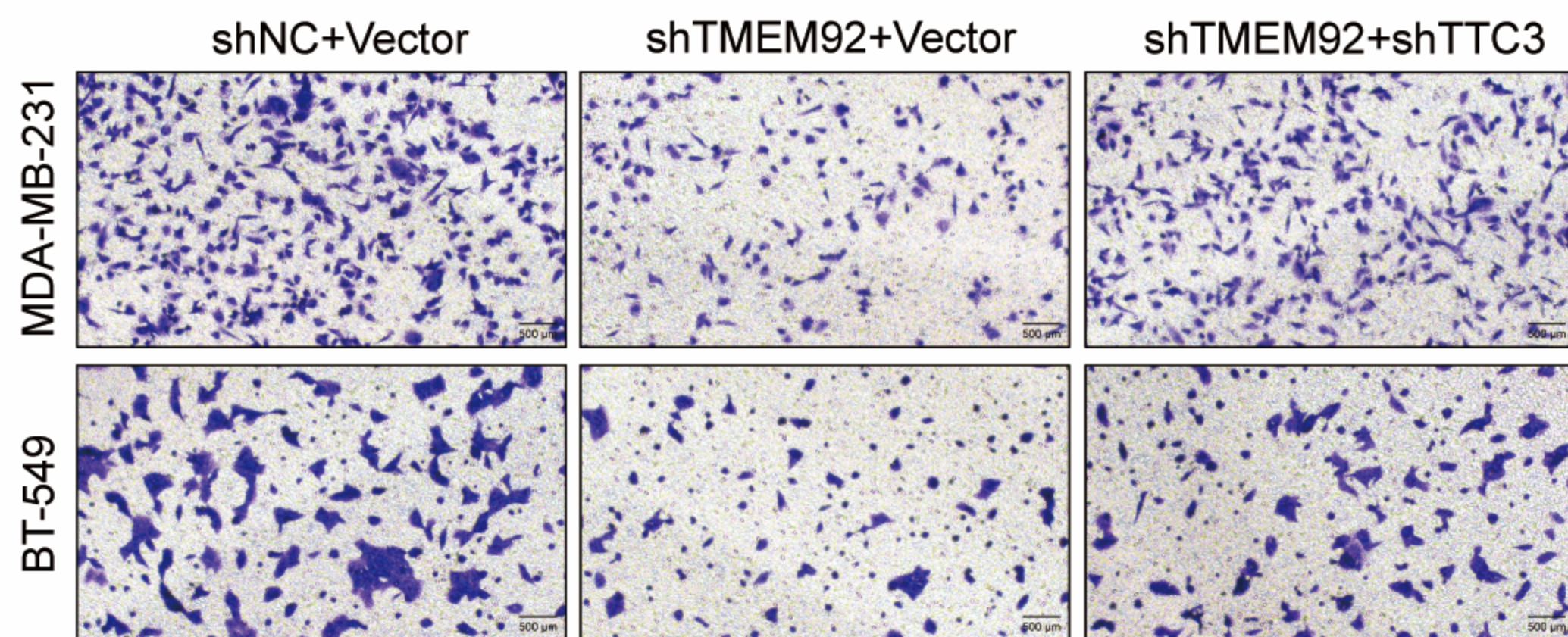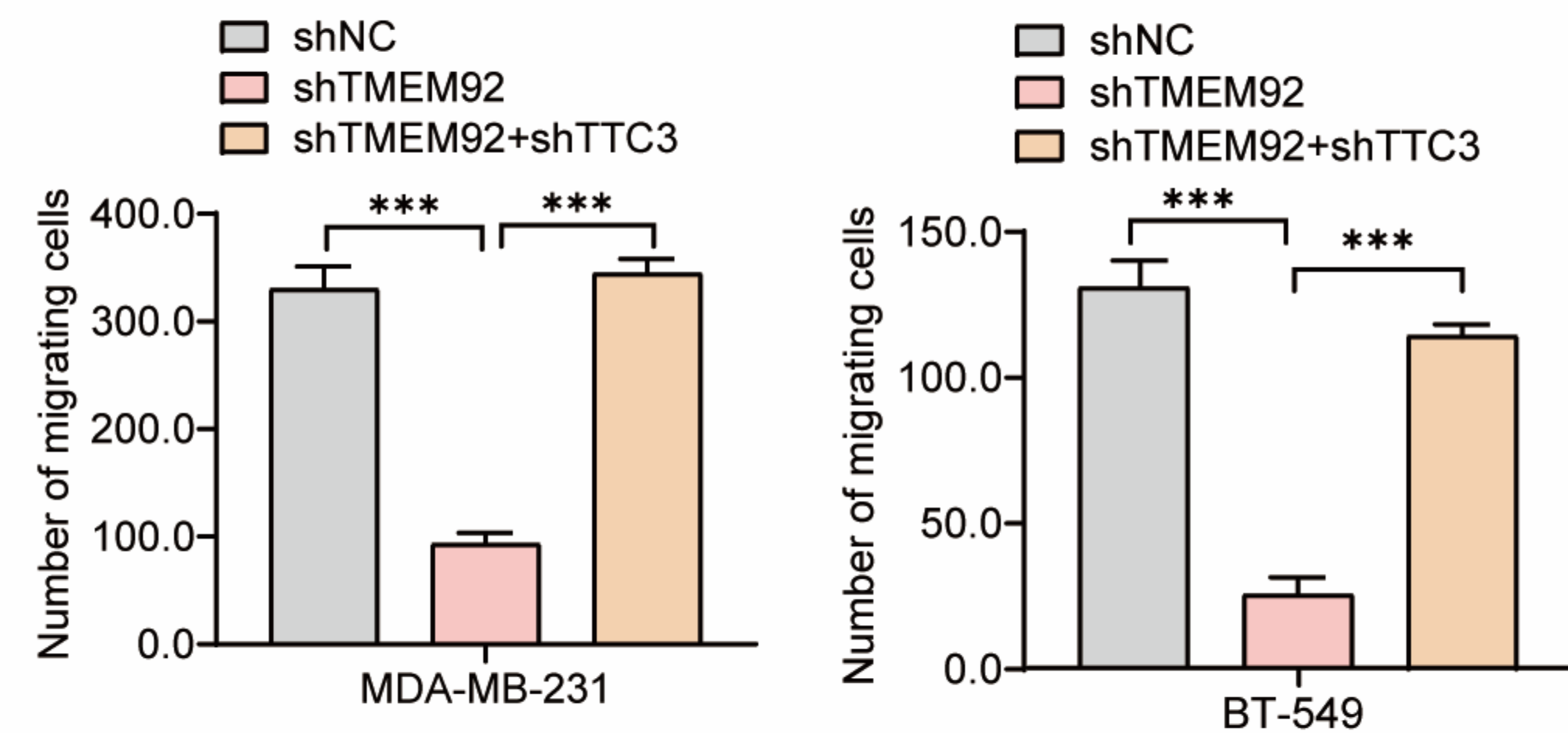

C

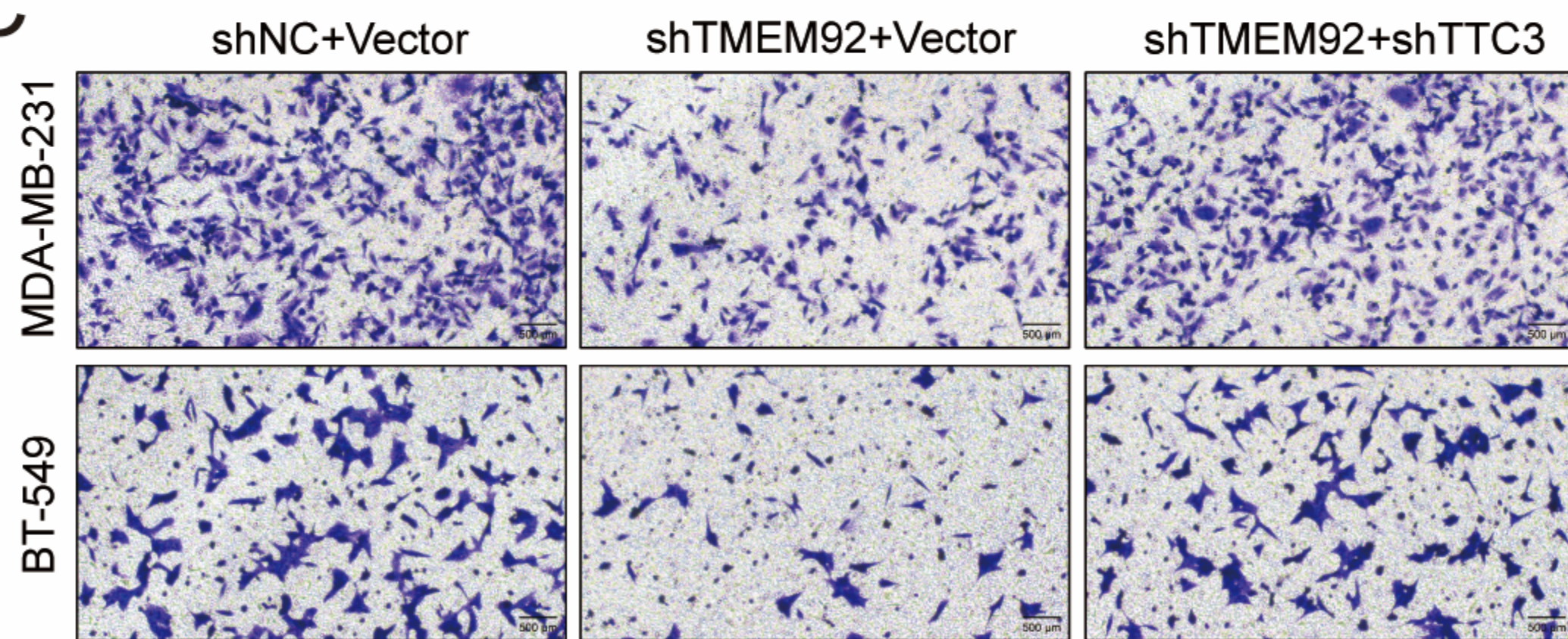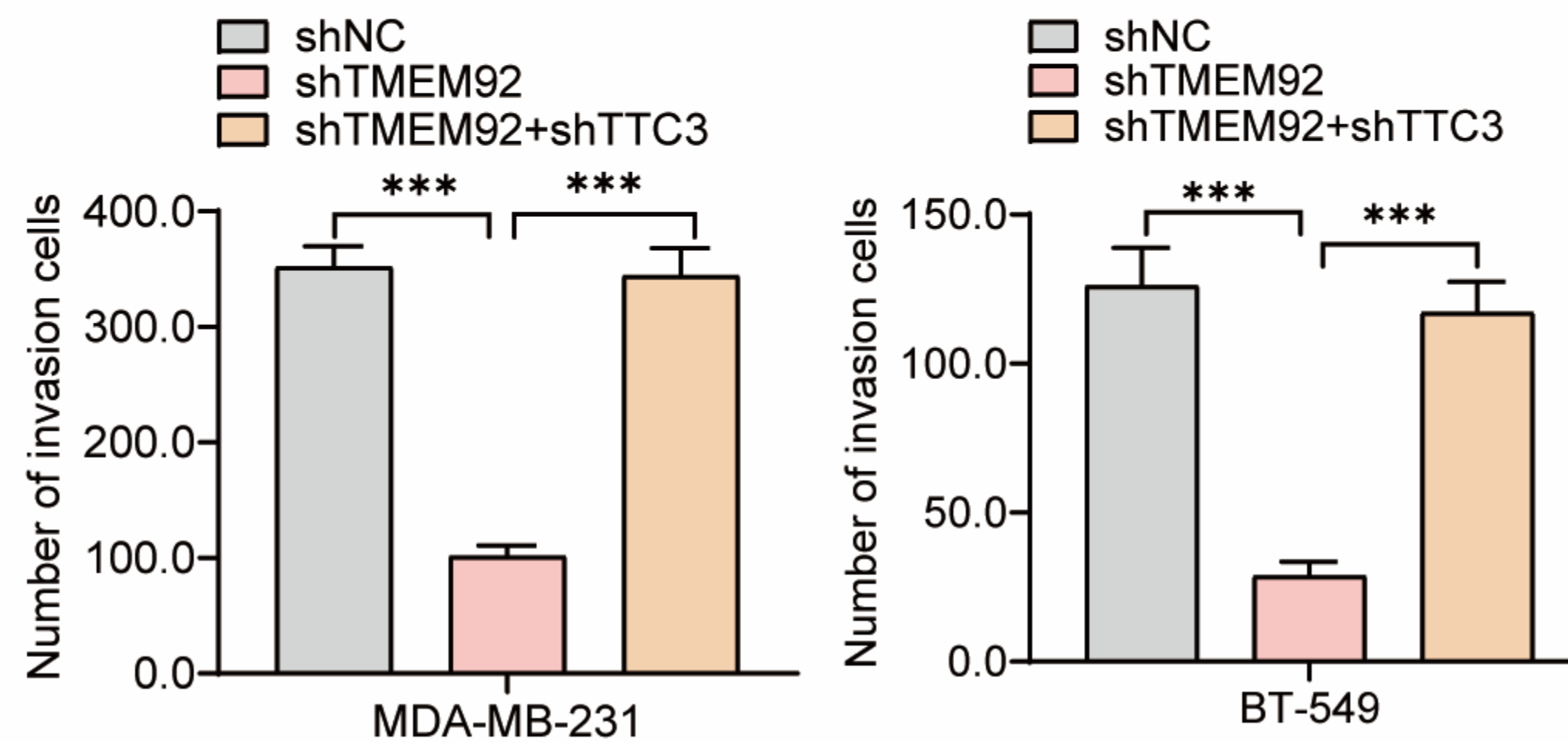

Supplement: Supplementary file 5 — Supporting Information [file CTM2-16-e70681-s009.pdf]

A

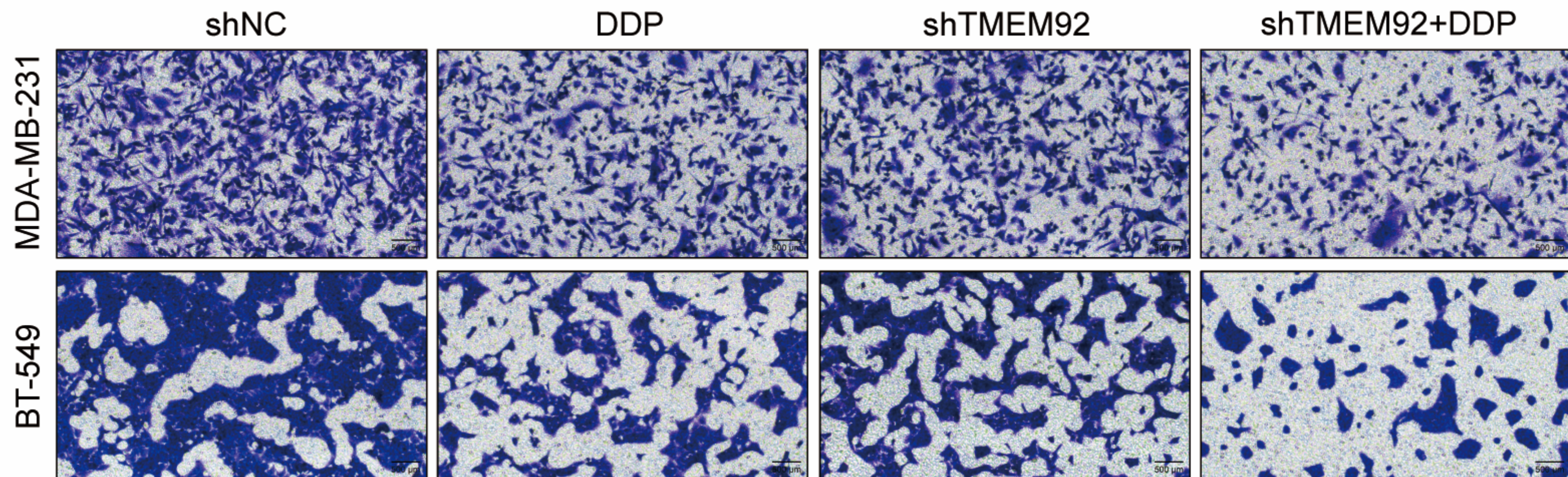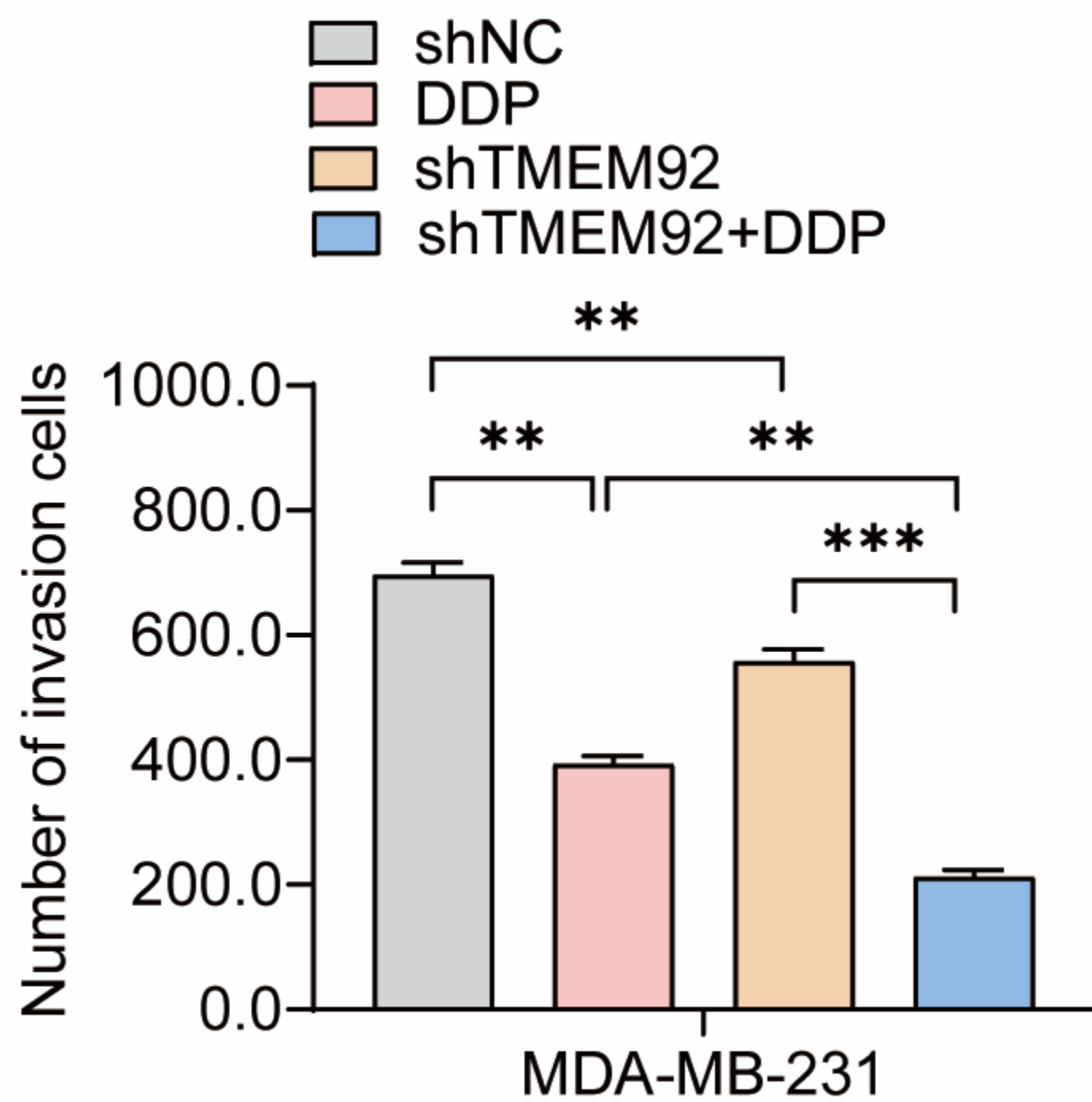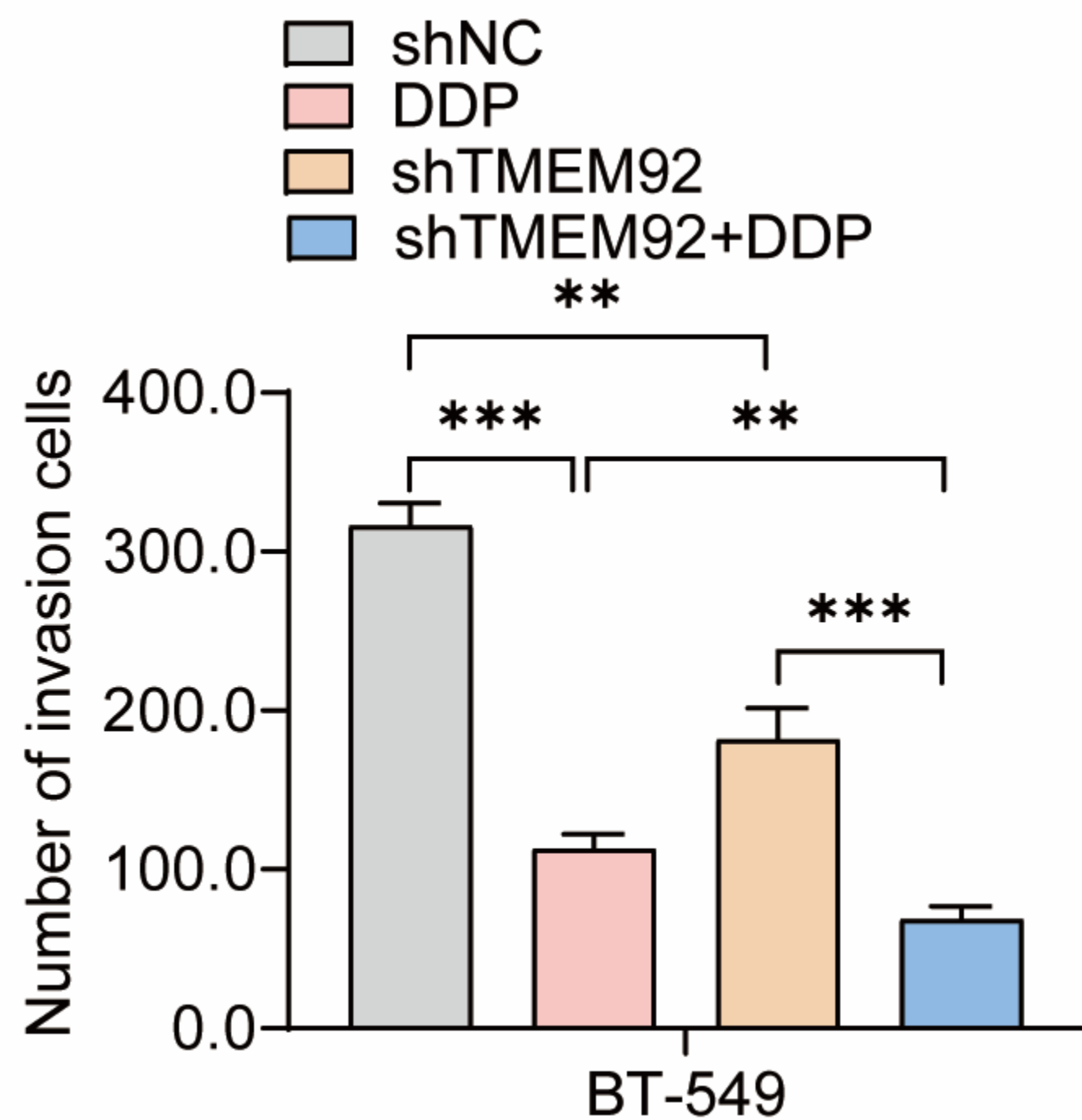

Supplement: Supplementary file 6 — Supporting Information [file CTM2-16-e70681-s010.pdf]

A

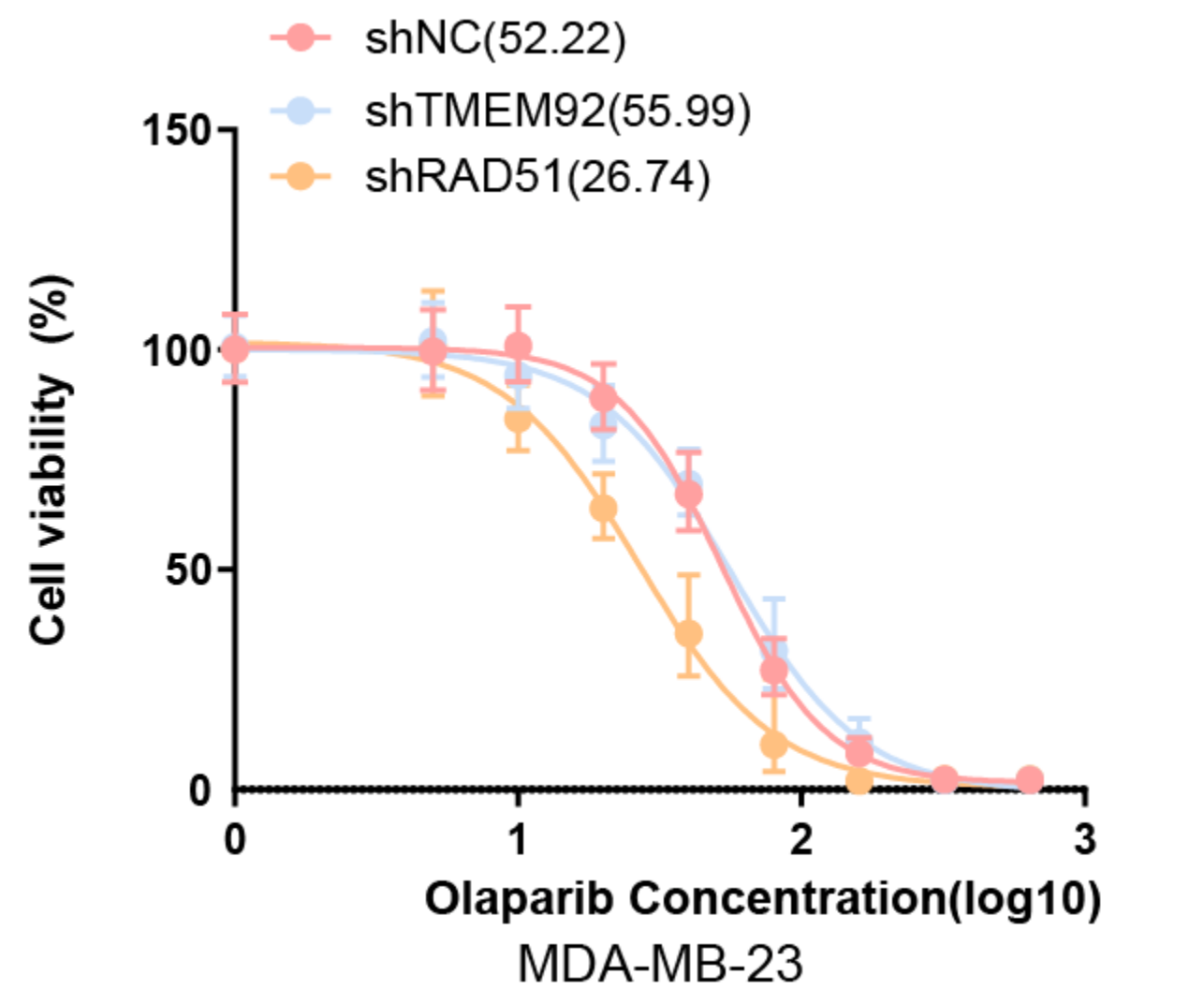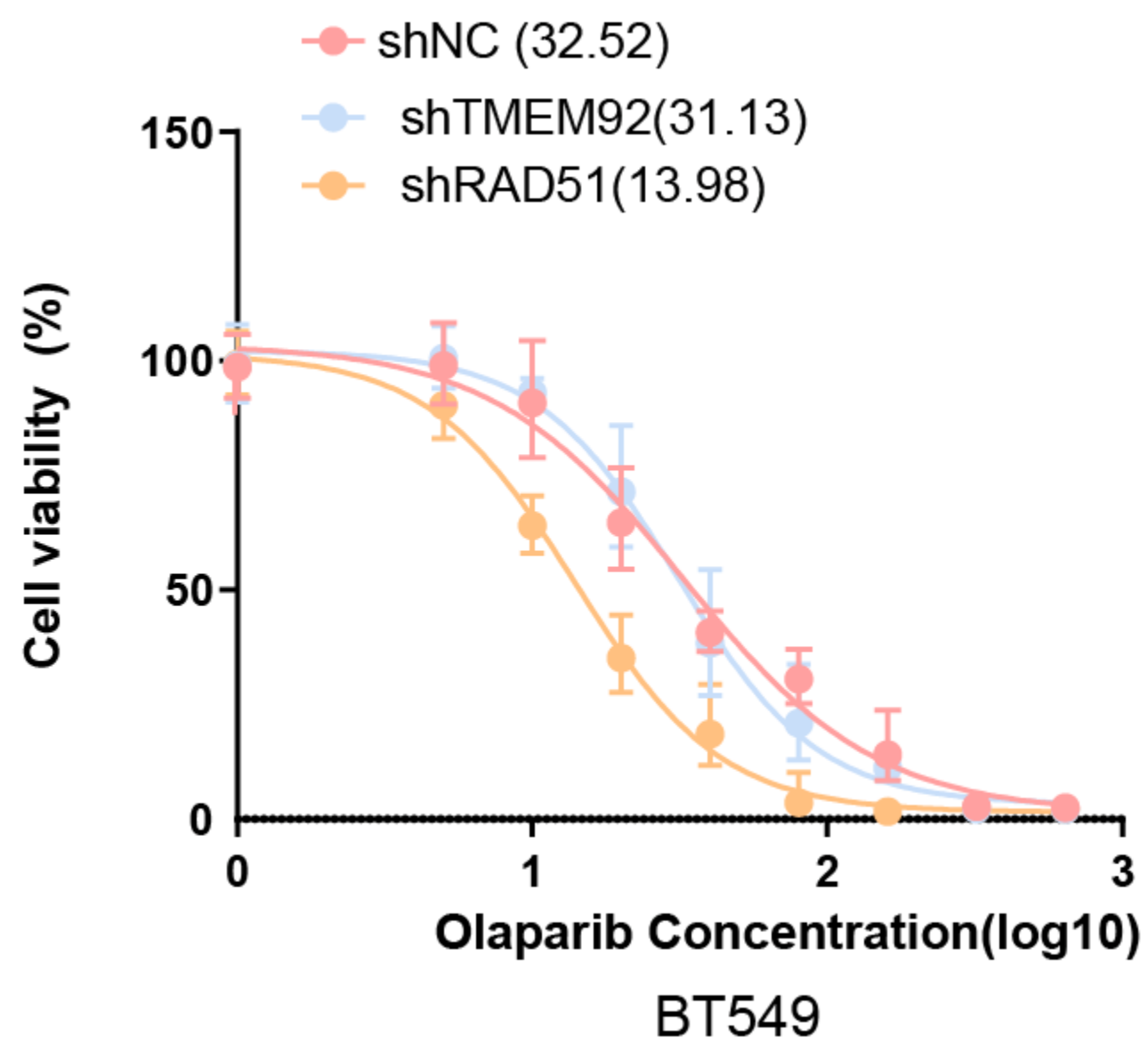

B

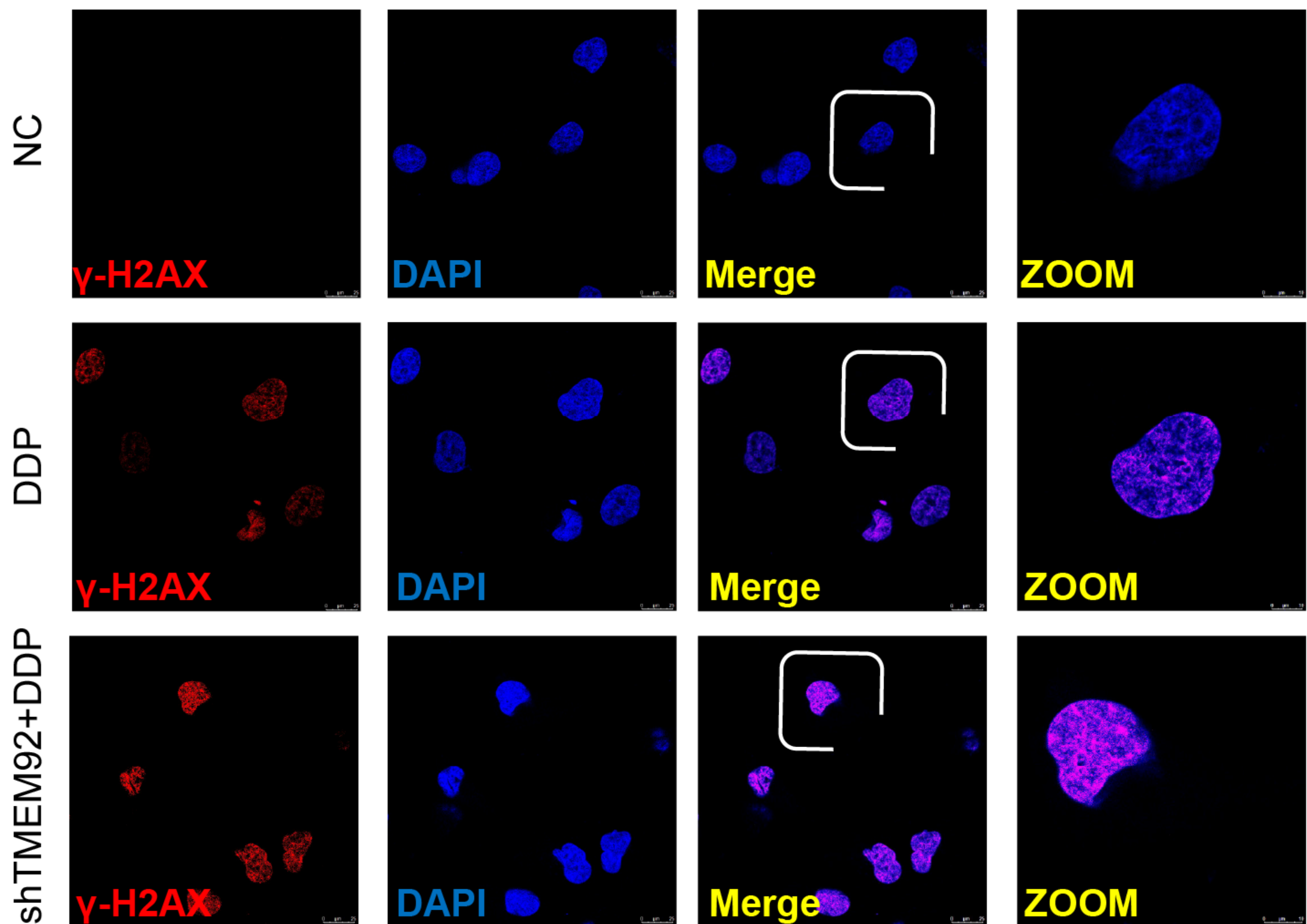

Supplement: Supplementary file 7 — Supporting Information [file CTM2-16-e70681-s003.pdf]

A

TMEM92-216791\_at

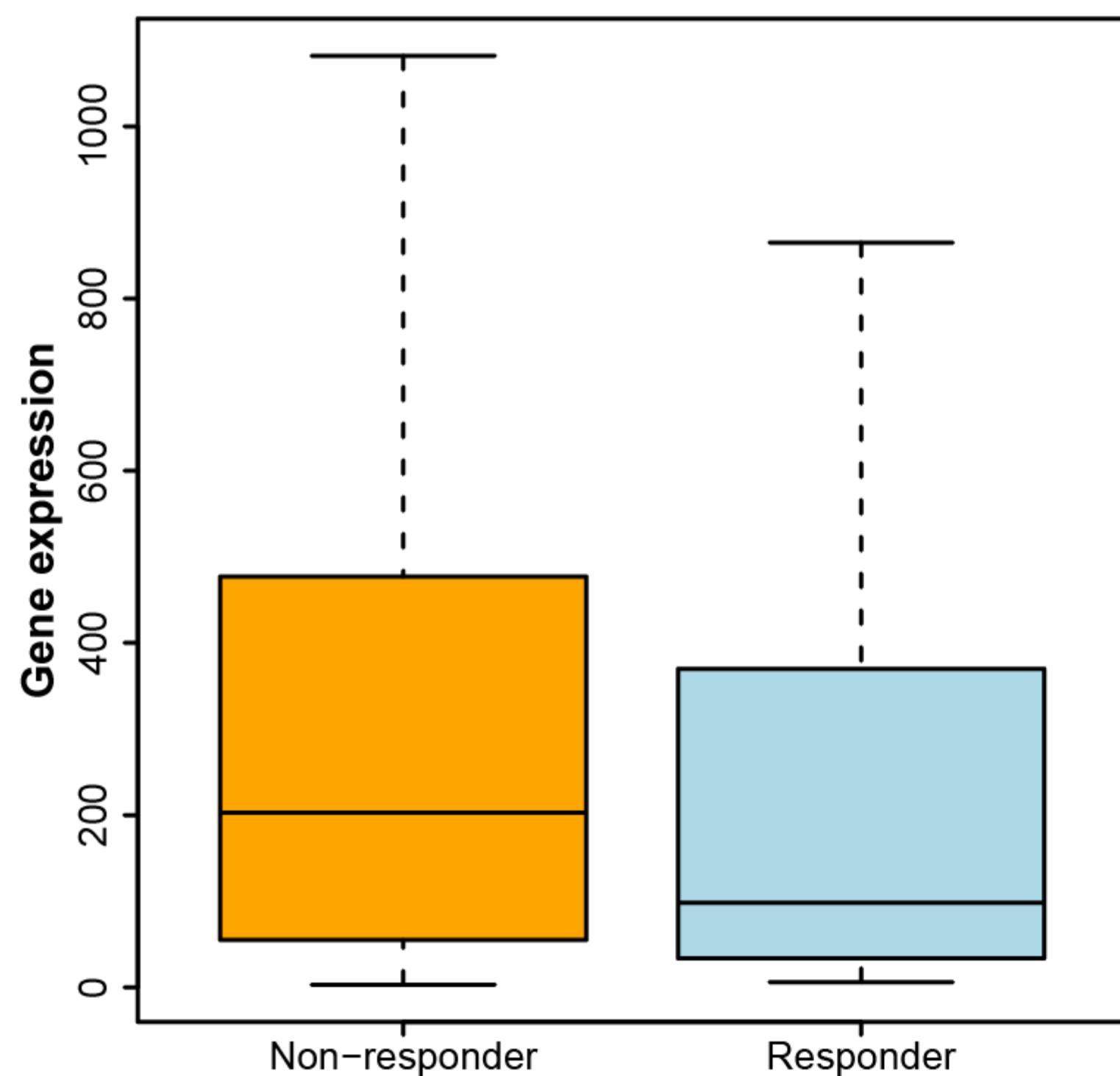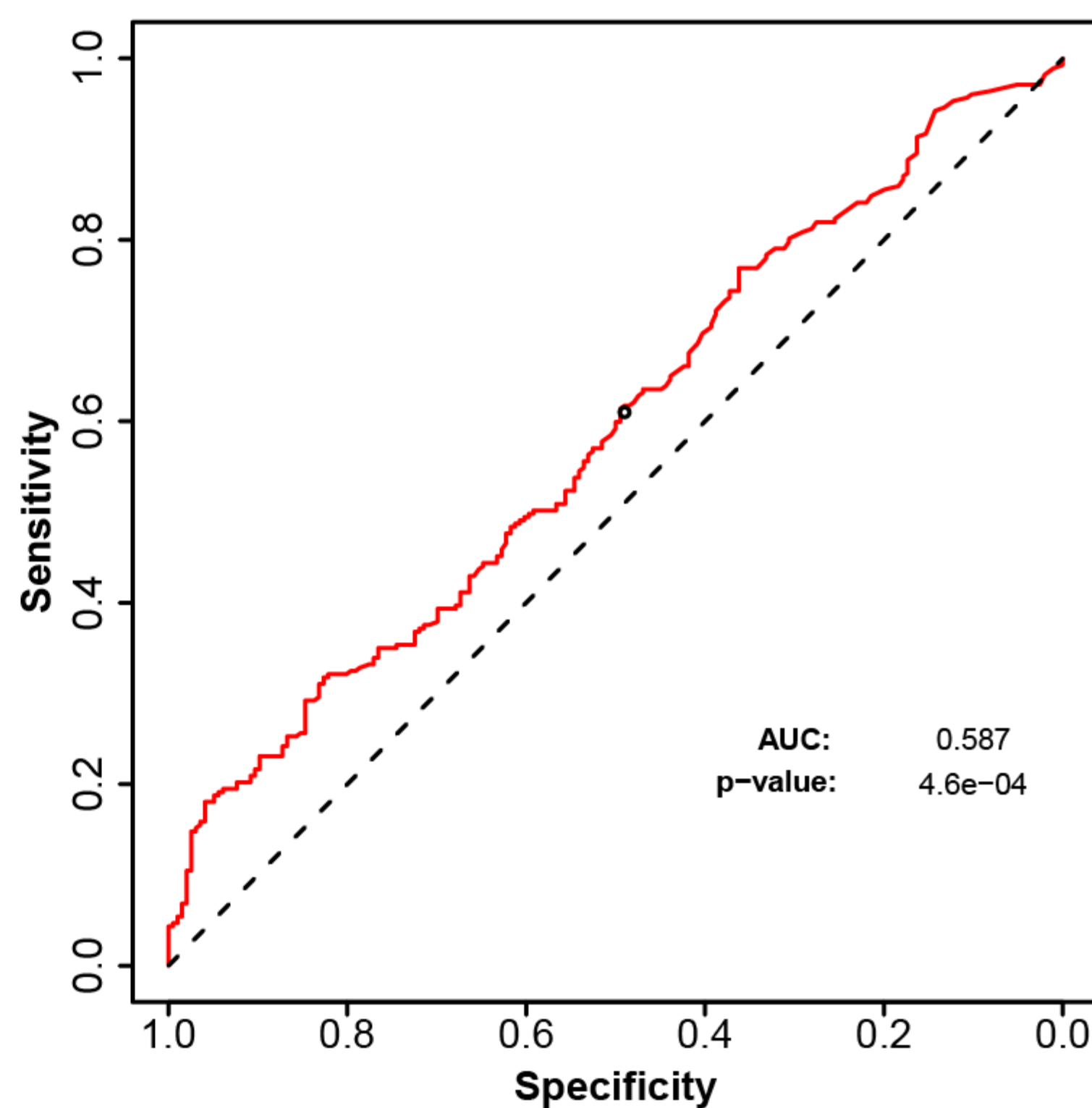

B

DDX3X-201211\_s\_at

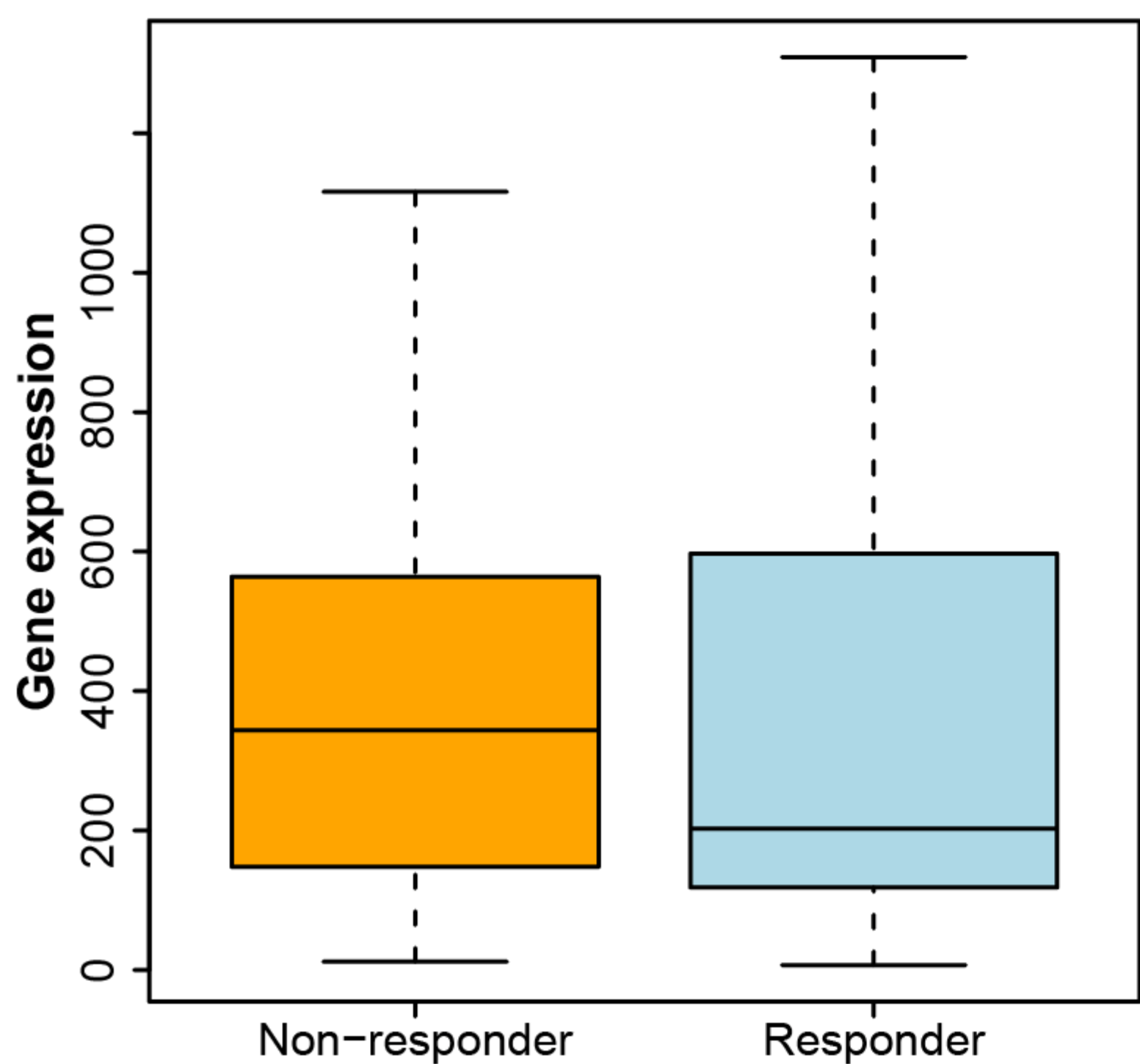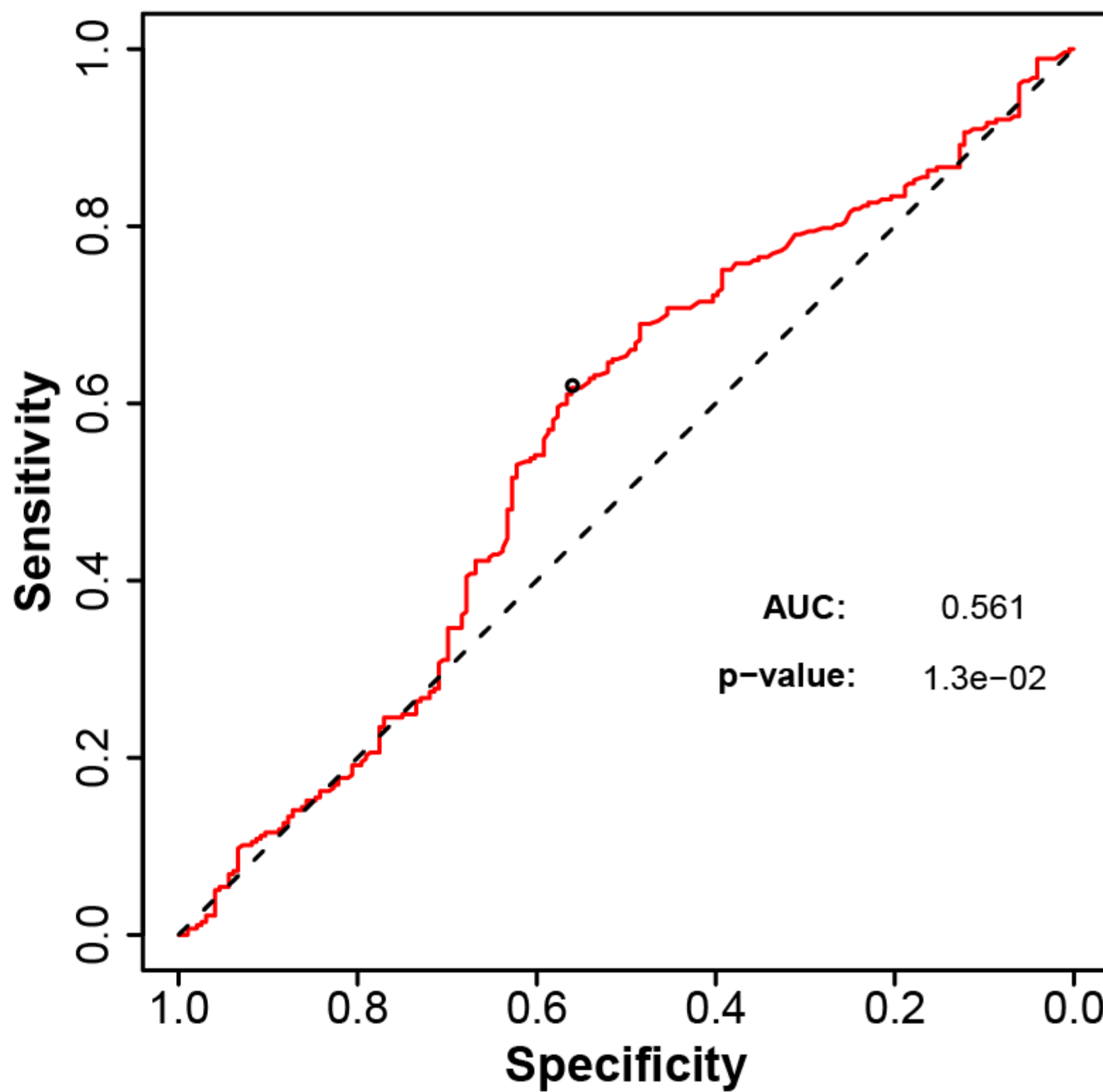

Supplement: Supplementary file 8 — Supporting Information [file CTM2-16-e70681-s001.pdf]
