# Supplementary material for: TMEM92 shields DDX3X from TTC3‐mediated degradation to confer chemoresistance in triple‐negative breast cancer
Source: Clin Transl Med. 2026 May 15;16(5):e70681. doi: 10.1002/ctm2.70681 (PMC13178151; doi:10.1002/ctm2.70681)
Supplement: Supplementary file 9 — Supporting Information [file CTM2-16-e70681-s005.docx]

**Supplementary Table 1** **ShRNA Sequences of TMEM92 and TTC3**

| Gene | Sequence |
| --- | --- |
| sh-TMEM92-1 | 5′-CAGCTTCAGGCCTGAAGAATA-3′ |
| sh-TMEM92-2 | 5′-GCTTCAGGCCTGAAGAATATA-3′ |
| sh-TMEM92-3 | 5′-CCTGCTATGTAGGAAACACTA-3′ |
| sh-TTC3-1 | 5′-CGAAGAGGACATAGTGGATTT-3′ |
| sh-TTC3-2 | 5′-GAAGAGGACTCACTGGATCTA-3 |
| sh-NC | 5′-TTCTCCGAACGTGTCACGT-3′ |

**Supplementary Table 2** **ShRNA Sequences of TMEM92, DDX3X and TTC3**

| Gene | Forward Primer（5'-3') | Reverse Primer（5'-3') |
| --- | --- | --- |
| TMEM92 | CATCCTGTCCGTCTTTTGCAT | GAAAGGGATACTCTGACCCTCT |
| DDX3X | ACGAGAGAGTTGGCAGTACAG | ATAAACCACGCAAGGACGAAC |
| TTC3 | GGACTATGTTATTCGCCACT | CTAATTTGGGCTCCACTTCT |
