# Supplementary material for: TMEM92 shields DDX3X from TTC3‐mediated degradation to confer chemoresistance in triple‐negative breast cancer
Source: Clin Transl Med. 2026 May 15;16(5):e70681. doi: 10.1002/ctm2.70681 (PMC13178151; doi:10.1002/ctm2.70681)
Supplement: Supplementary file 10 — Supporting Information [file CTM2-16-e70681-s004.docx]

| Clinical stage | Mean IHC score ± SEM |
| --- | --- |
| Paracancerous tissue | 0.767 ± 0.145 |
| Stage I | 1.600 ± 0.208 |
| Stage II | 1.767 ± 0.088 |
| Stage III | 2.267 ± 0.145 |
| Stage IV | 4.000 ± 0.289 |

Supplementary Table S1. TMEM92 IHC H-scores across clinical stages
