# Supplementary material for: TMEM92 shields DDX3X from TTC3‐mediated degradation to confer chemoresistance in triple‐negative breast cancer
Source: Clin Transl Med. 2026 May 15;16(5):e70681. doi: 10.1002/ctm2.70681 (PMC13178151; doi:10.1002/ctm2.70681)
Supplement: Supplementary file 11 — Supporting Information [file CTM2-16-e70681-s008.docx]

**Fig. S1 Validation of knockdown and overexpression constructs.** A, B. The qRT-PCR and Western blot validation of TMEM92 knockdown (N = 3 biological replicates for qRT-PCR, mean ± SD; representative WB from N = 3 independent experiments). C, D. Validation of TMEM92 overexpression (N = 3 biological replicates for qRT-PCR, mean ± SD; representative WB from N = 3 independent experiments). E, F. Validation of DDX3X overexpression (N = 3 biological replicates for qRT-PCR, mean ± SD; representative WB from N = 3 independent experiments). G, H. Validation of TTC3 overexpression (N = 3 biological replicates for qRT-PCR, mean ± SD; representative WB from N = 3 independent experiments). I, J. Validation of TTC3 knockdown (N = 3 biological replicates for qRT-PCR, mean ± SD; representative WB from N = 3 independent experiments). (***p* < 0.01, ****p* < 0.001).

**Fig. S2 TMEM92 promotes TNBC cell migration and invasion independently of ER, PR, and HER2 expression.** A. Western blot analysis showing that TMEM92 knockdown does not alter the protein expression levels of ER, PR, or HER2 in MDA-MB-231 and BT-549 cells. GAPDH served as a loading control (representative WB from N = 3 independent experiments). B, C. Transwell migration and invasion assays demonstrating that TMEM92 overexpression significantly enhances migratory and invasive capacities of MDA-MB-231 and BT-549 cells (representative images; N = 3 independent experiments, mean ± SD).D.Validation of TMEM92 genetic ablation. RT-qPCR (left) and Western blot (right) analyses confirm the successful knockout of TMEM92 using CRISPR/Cas9 (two independent knockout clones, clone #1 and clone #2) in MDA-MB-231 and BT-549 cells. GAPDH served as a loading control for Western blot. E, F. Genetic ablation of TMEM92 using CRISPR/Cas9 (two independent knockout clones) markedly suppresses cell migration (D) and invasion (E), while re-expression of TMEM92 in knockout cells restores these phenotypes (representative images; N = 3 independent experiments, mean ± SD).

**Fig. S3 TMEM92 enhances TNBC cell proliferation and confers resistance to DDP-induced apoptosis.** A. Colony formation assays showing that TMEM92 overexpression significantly promotes clonogenic growth in MDA-MB-231 and BT-549 cells, whereas TMEM92 knockout suppresses colony formation; re-expression of TMEM92 in knockout cells restores clonogenic capacity (representative images; N = 3 independent experiments, mean ± SD). B. CCK-8 cell proliferation assays indicating accelerated growth in TMEM92-overexpressing cells compared with controls (N = 3 biological replicates, mean ± SD). C. Flow cytometric analysis of Annexin V/PI staining showing that TMEM92 overexpression reduces basal apoptosis, whereas TMEM92 knockout significantly increases apoptotic rates in TNBC cells; these effects are partially reversed by TMEM92 re-expression (representative plots; N = 3 independent experiments, mean ± SD). D. Dose–response curves of DDP treatment demonstrating that TMEM92 knockout markedly sensitizes TNBC cells to DDP, while TMEM92 re-expression restores DDP resistance (N = 3 independent experiments, mean ± SD). Statistical significance was determined as indicated (*p < 0.05, **p < 0.01, ***p < 0.001).

**Fig. S4 TMEM92-mediated regulation of DDX3X is independent of the lysosomal pathway.** A. Western blot analysis of DDX3X in control and shTMEM92 cells treated with or without chloroquine (CQ)(representative WB from N = 3 independent experiments).

**Fig. S5 Knockdown of TTC3 rescues malignant phenotypes suppressed by TMEM92 depletion.** A. Cell proliferation rescue assay (N = 3 biological replicates, mean ± SD). B, C. Transwell migration and invasion rescue assays (representative images; N = 3 independent experiments, mean ± SD). (**p* < 0.05, ***p* < 0.01, ****p* < 0.001).

**Fig. S6 Knockdown of TMEM92 synergizes with DDP to inhibit TNBC cell invasion.** A. Transwell invasion assay of control and shTMEM92 cells treated with or without DDP (representative images; N = 3 independent experiments, mean ± SD). (***p* < 0.01, ****p* < 0.001).

**Fig. S7. TMEM92 depletion does not induce a typical HR-deficient phenotype but enhances cisplatin-induced DNA damage.**A. Cell viability curves of MDA-MB-231 and BT549 cells treated with Olaparib under the indicated conditions (shNC, shTMEM92, and shRAD51). TMEM92 knockdown did not increase Olaparib sensitivity compared with shRAD51. IC50 values are shown in parentheses.B. Representative immunofluorescence images of γ-H2AX staining in MDA-MB-231 cells under the indicated treatments (NC, DDP, and shTMEM92 + DDP). γ-H2AX is shown in red and nuclei are stained with DAPI (blue). Merged and enlarged images are shown on the right. TMEM92 knockdown increased γ-H2AX accumulation after DDP treatment. Scale bars, 25 μm (main images) and 10 μm (zoomed images).

**Fig. S8. Clinical association of TMEM92 and DDX3X expression with chemotherapy response in TNBC.**A.Box plot and ROC curve showing the association between TMEM92 expression and chemotherapy response in TNBC patients from the ROC Plotter database. TMEM92 expression was higher in non-responders than in responders, and ROC analysis showed a significant association with pathological response (AUC = 0.587, P = 4.6 × 10^-4^).B.Box plot and ROC curve showing the association between DDX3X expression and chemotherapy response. DDX3X expression was also significantly associated with pathological response (AUC = 0.561, P = 1.3 × 10^-2^), although the discriminative ability was relatively limited.These data suggest that elevated TMEM92 and DDX3X expression is associated with chemotherapy non-response in TNBC.
