# Supplementary material for: TMEM92 shields DDX3X from TTC3‐mediated degradation to confer chemoresistance in triple‐negative breast cancer
Source: Clin Transl Med. 2026 May 15;16(5):e70681. doi: 10.1002/ctm2.70681 (PMC13178151; doi:10.1002/ctm2.70681)
Supplement: Supplementary file 2 — Supporting Information [file CTM2-16-e70681-s007.pdf]

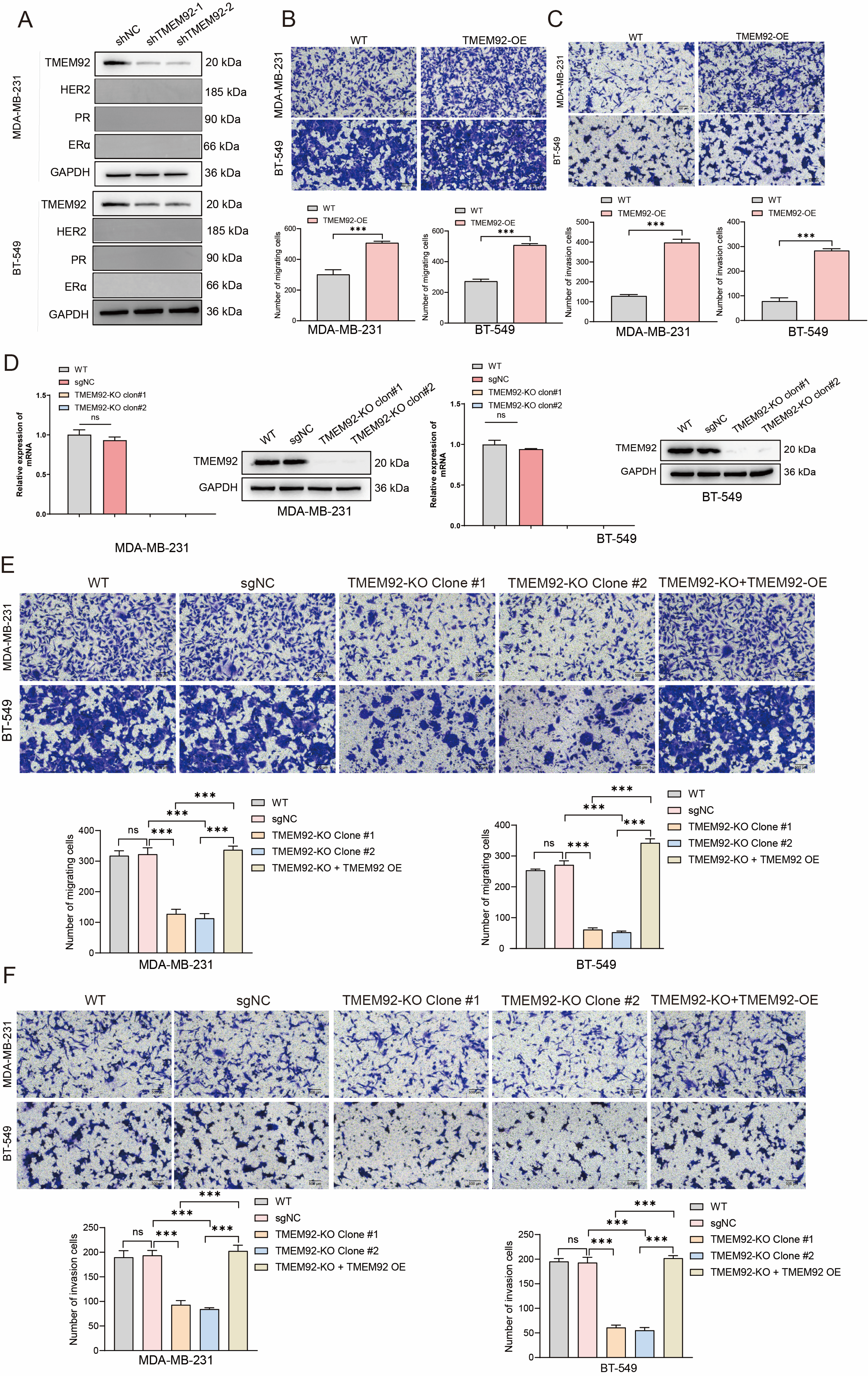

A
shNC
shTMEM92-1
shTMEM92-2
MDA-MB-231
TMEM92
20 kDa
HER2
185 kDa
PR
90 kDa
ERα
66 kDa
GAPDH
36 kDa
BT-549
TMEM92
20 kDa
HER2
185 kDa
PR
90 kDa
ERα
66 kDa
GAPDH
36 kDa
B
WT
TMEM92-OE
MDA-MB-231
BT-549
WT
TMEM92-OE
Number of migrating cells
***
MDA-MB-231
BT-549
C
WT
TMEM92-OE
MDA-MB-231
BT-549
Number of invasion cells
***
MDA-MB-231
BT-549
D
WT
sgNC
TMEM92-KO clon#1
TMEM92-KO clon#2
Relative expression of mRNA
ns
MDA-MB-231
TMEM92
20 kDa
GAPDH
36 kDa
BT-549
E
WT
sgNC
TMEM92-KO Clone #1
TMEM92-KO Clone #2
TMEM92-KO+TMEM92-OE
MDA-MB-231
BT-549
TMEM92-KO + TMEM92 OE
Number of migrating cells
ns
***
MDA-MB-231
BT-549
F
WT
sgNC
TMEM92-KO Clone #1
TMEM92-KO Clone #2
TMEM92-KO+TMEM92-OE
MDA-MB-231
BT-549
TMEM92-KO + TMEM92 OE
Number of invasion cells
ns
***
MDA-MB-231
BT-549
